# A detoxification pathway initiated by a nuclear receptor TcHR96h in *Tetranychus cinnabarinus* (Boisduval)

**DOI:** 10.1371/journal.pgen.1010911

**Published:** 2023-09-14

**Authors:** Xiang Wen, Kaiyang Feng, Juan Qin, Peng Wei, Peng Cao, Youjun Zhang, Zhiguang Yuchi, Lin He

**Affiliations:** 1 Key Laboratory of Entomology and Pest Control Engineering, College of Plant Protection, Southwest University, Chongqing, China; 2 Key Laboratory of Agricultural Biosafety and Green Production of Upper Yangtze River, Ministry of Education, Chongqing, China; 3 National Citrus Engineering Research Center, Southwest University, Chongqing, China; 4 Tianjin Key Laboratory for Modern Drug Delivery & High-Efficiency, Collaborative Innovation Center of Chemical Science and Engineering, School of Pharmaceutical Science and Technology, Tianjin University, Tianjin, China; 5 Key Laboratory of Drug Targets and Drug Leads for Degenerative Diseases, Affiliated Hospital of Integrated Traditional Chinese and Western Medicine, Nanjing University of Chinese Medicine, Nanjing, China; 6 Department of Plants and Crops, Institute of Vegetables and Flowers, Chinese Academy of Agricultural Sciences, Beijing, China; University of Kentucky, UNITED STATES

## Abstract

Understanding the mechanism of detoxification initiation in arthropods after pesticide exposure is crucial. Although the identity of transcription factors that induce and regulate the expression of detoxification genes in response to pesticides is beginning to emerge, whether transcription factors directly interact with xenobiotics is unclear. The findings of this study revealed that a nuclear hormone receptor, *Tetranychus cinnabarinus* hormone receptor (HR) *Tc*HR96h, regulates the overexpression of the detoxification gene *TcGSTm02*, which is involved in cyflumetofen resistance. The nuclear translocation of *Tc*HR96h increased after cyflumetofen exposure, suggesting direct binding with cyflumetofen. The direct binding of *Tc*HR96h and cyflumetofen was supported by several independent proteomic assays that quantify interactions with small molecules. Together, this study proposes a model for the initiation of xenobiotic detoxification in a polyphagous agricultural pest. These insights not only provide a better understanding of the mechanisms of xenobiotic detoxification and metabolism in arthropods, but also are crucial in understanding adaptation in polyphagous herbivores.

## Author summary

Phytophagous arthropods possess a powerful system of detoxification that involves several major metabolic detoxification gene families. What is currently clear is how detoxification enzymes interact with chemical molecules. However, it remains elusive how insects sense the xenochemicals and signal to activate the downstream detoxification system when faced with pesticide exposure. The present study identified a xenobiotic sensor that initiates a detoxification system and a novel pathway in the detoxification of cyflumetofen in a representative mite species. The information will contribute a deeper insight into the whole process of arthropods even animal detoxification and metabolism.

## Introduction

Pesticides, including insecticides and acaricides, are an essential component of global pest management strategies against crop pests [1,2]. However, the development of resistance is a serious threat to sustainable food production. Pesticides, and toxicants in general, induce the expression of detoxification genes and production of metabolic enzymes and transporters [3,4]. Constitutive changes in the expression of detoxification genes have been associated with resistance in many cases. However, how arthropods regulate the expression of detoxification genes upon direct exposure to exogenous toxicants is unclear. This hinders a comprehensive understanding of xenobiotic detoxification, a crucial component for adaptation to plant toxins and synthetic insecticides, especially in polyphagous crop pests.

In vertebrates and invertebrates, three major transcription factor (TF) families have been implicated in the xenobiotic response to toxicants: the basic leucine zipper (bZIP) family, the basic helix-loop-helix/Per–ARNT–Sim family, and nuclear receptors [5–9]. In insects, the bZIP factor CncC plays a vital role in detoxification. For example, the CncC pathway regulates *CYP6BQ9* expression in response to pyrethroids in *Tribolium castaneum* [10]. The aromatic hydrocarbon receptor AhR plays a role not only in the morphological development of organs, but also in response to fungicides and bactericides [11]. In arthropods, the AhR orthologue *spineless* modulates basal expression from the *CYP6B1* promoter in a ligand-independent manner. Moreover, it attenuates subsequent responses to planar aryl hydrocarbons (benzo[α]pyrene) and allelochemicals (xanthotoxin) in *Drosophila* [6]. Studies in various insect species have shown that TFs, such as CncC or AhR, can up-regulate the expression of detoxification genes, including GSTs, which are involved in insecticide resistance [12,13]. Although many studies have provided evidence that TFs regulate gene expression upon pesticide exposure in arthropods, scant evidence exists that xenobiotics directly activate TFs that act as xenosensors.

Among the three TF superfamilies, only the nuclear hormone receptor (NHR) family, which is implicated in various physiological functions of development, homeostasis, and metabolism, was predicted to be ligand-activated [14]. Vertebrate nuclear receptors, such as the steroid and xenobiotic receptor (SXR in humans; PXR in mice) and the constitutive androstane receptor (CAR), have been extensively investigated [15,16]. Studies have shown that PXR and CAR respond to a wide range of xenobiotics and regulate the expression of overlapping sets of genes, including genes encoding phase I/II detoxifying enzymes, such as cytochrome P450 and GST [17]. Insect HR96 is the single orthologue of CAR and PXR [18], whereas several paralogs have been described in mites [19]. In *Drosophila*, DHR96 was shown to regulate phenobarbital-inducible DDT-resistance genes, including *CYP6B1* and *CYP6G1* [20]. In *Daphnia pulex*, the HR96 orthologue reportedly responds to a wide range of endobiotics and xenobiotics [21]. In the two-spotted spider mite (*Tetranychus urticae*), one of the HR96 paralogs, *tetur36g00260* was reported to be associated with gene expression patterns related to host plant adaptation and pesticide resistance [22]. Together, these studies suggest that in insects and mites, HR96 plays an important role in response to xenobiotics. Clear evidence that HR96 can bind xenobiotics directly remains elusive. The findings of this study could bridge the gap between pesticide exposure and xenobiotic detoxification, thus improving our understanding of the initiation of detoxification in arthropods.

The carmine spider mite (*Tetranychus cinnabarinus*) and *T*. *urticae* are notorious pests that cause enormous economic loss [23,24]. Studies have revealed that gene families involved in the digestion, detoxification, and transportation of xenobiotics are often expanded in *T*. *urticae* compared with other arthropods [25–27]. These include novel metabolic activities, such as those acquired after horizontal gene transfer [28]. Thus, spider mites provide a promising and robust experimental system for the study of detoxification upon insecticide exposure. Chemical control with synthetic acaricides is a crucial component of integrated pest management programs, which have cost approximately >900 million euros [29]. Cyflumetofen is a novel benzoyl acetonitrile acaricide that is highly effective against spider mites belonging to the genera *Tetranychus* and *Panonychus* [30]. Cyflumetofen, an inhibitor of complex II in the mitochondrial electron transport chain, is widely used in 15 countries for mite control [31]. Because complex II inhibitors represent a novel mode of action for arthropod control, elucidating the mechanism of cyflumetofen detoxification and resistance is crucial and has recently become a topic of focus of toxicological studies in spider mites. Studies have suggested that, next to target-site resistance mutations [32], detoxification genes, such as GST, play a crucial roles in cyflumetofen detoxification [33–36]. However, the mechanisms of overexpression of this GST, is yet to be resolved.

To assess whether HR96 is involved in the overexpression of GST in a *T*. *cinnabarinus* cyflumetofen-resistant (CyR) strain, the relative expression of all eight HR96 genes (*TcHR96a*–*TcHR96h*) was characterized. The findings revealed that *TcHR96h* expression is 18.2-fold higher in the resistant strain. RNAi-mediated silencing of *TcHR96h* confirmed its role in mediating cyflumetofen detoxification. Although silencing *TcHR96h* decreased the expression of eight out of twelve GST genes belonging to class mu, the largest effect was obtained for *TcGSTm02*, which was chosen as the target for this study. The findings showed that *TcHR96h* can be activated by cyflumetofen through direct binding, after which it translocates to the nucleus where it regulates *TcGSTm02* expression through promotor interactions. Together, these results provide the first evidence that the initiation and regulation of xenobiotic detoxification in mites is mediated by xenobiotic binding to nuclear receptor HR96.

## Results

### *TcHR96h* expression is higher in strain CyR

**Fig 1:**
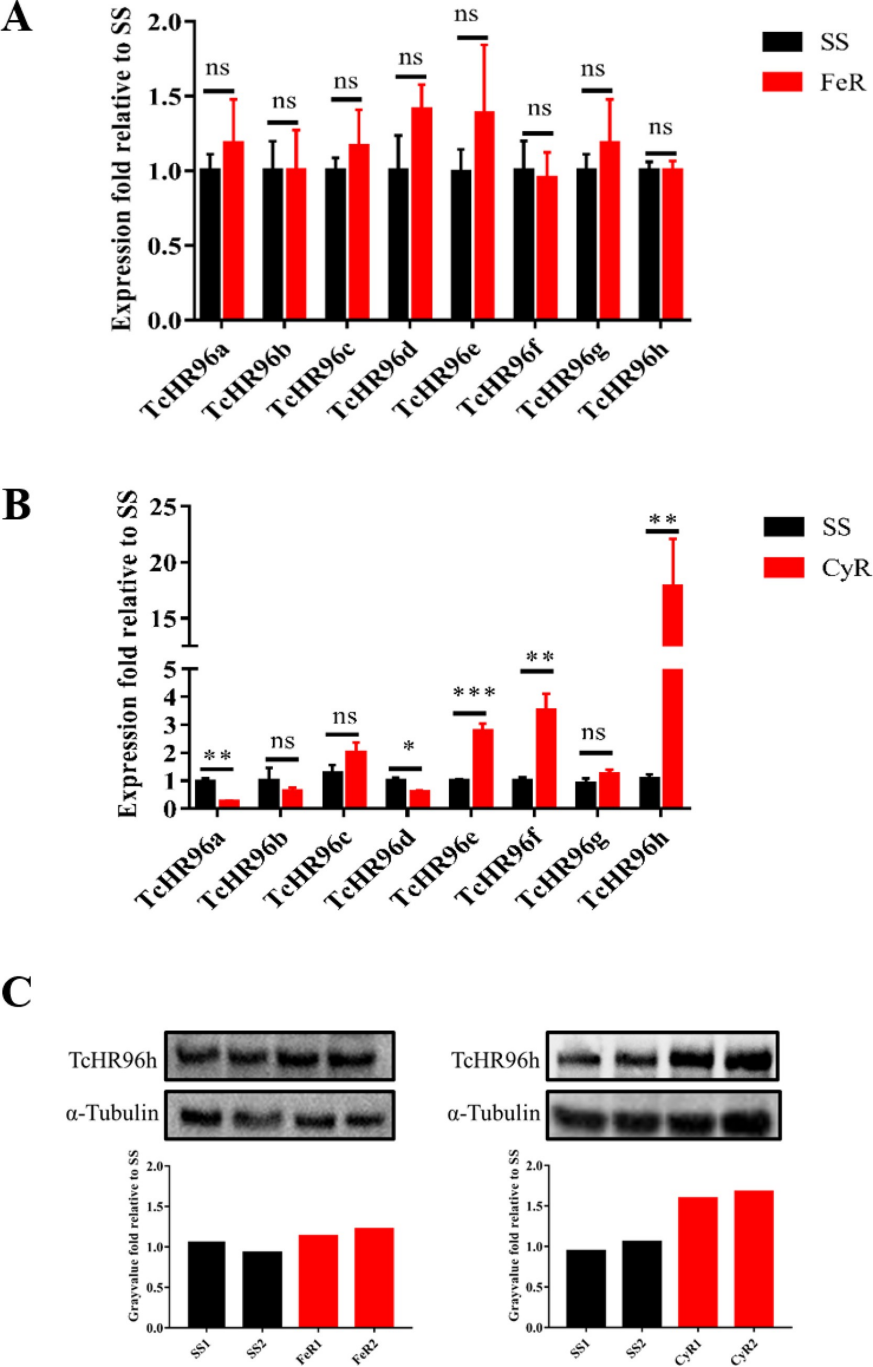
Relative expression of canonical HR96 in different strains. The relative expression of the eight canonical *HR96* mRNAs in the fenpropathrin-resistant (FeR) (A) and cyflumetofen-resistant (CyR) (B) strains compared with the susceptible strain (SS) was determined by qPCR (qPCR: n = 3, mean ± SE, asterisk represents significant difference (FeR/CyR compared with SS), *P < 0.05, **P < 0.01, ***P < 0.001, two-tailed Student’s *t*-test). (C) Relative expression of *Tc*HR96h in SS, FeR, and CyR strains is evaluated by western blotting (upper row) and quantitative estimation of band intensity by densitometry using ImageJ software and normalized to α-tubulin (graph) are presented (lower row). The rabbit polyclonal antibody against recombinant *Tc*HR96h was generated and diluted with Tris-buffered saline-Tween at ratio of 1:8000 before use; α-tubulin (1:5000) was used as control.

The expression of HR96 family genes was examined by analyzing published transcriptomic data. The results indicate that 10 and 34 of HR96 genes were overexpressed in CyR and YN-CyR, respectively, suggesting a strong association between cyflumetofen resistance and HR96 overexpression (S1 Fig). Additionally, Quantitative PCR was performed to assess the expression levels of the eight canonical HR96 paralogs in SS, CyR, and FeR strains of *T*. *cinnabarinus*. Although the difference in expression between SS and FeR strains was insignificant (Fig 1A), the expression of *TcHR96h*, *TcHR96e*, and *TcHR96f* was significantly higher in strain CyR. *TcHR96h* showed the highest overexpression, reaching 18.2-fold that of SS (Fig 1B). Western blotting confirmed increased expression (1.68 fold) (Fig 1C).

### Reduced *TcHR96h* expression alters cyflumetofen toxicity

To examine the role of *TcHR96h* overexpression in strain CyR, RNAi was used to knock down *TcHR96h* expression in adult mites, and the change in susceptibility against cyflumetofen was evaluated using bioassays. Using a specific dsRNA (S3A Fig), silencing was determined to be 59% (± 5%) in SS (S3B Fig), which decreased the LC_50_ value of cyflumetofen from 2.9 mg/L (95%, CL: 2.4–3.6 mg/L) to 1.5 mg/L (95%, CL: 1.2–1.8 mg/L) (Table 1). Similar results were obtained with strain CyR, where silencing efficiency reached 80% (± 13%), reducing the LC_50_ value of cyflumetofen from 235 mg/L (95%, CL: 210–254 mg/L) to 68 mg/L (95%, CL: 38–90 mg/L) (Table 1). Similarly, the LC_50_ value of *T*. *cinnabarinus* to fenpropathrin was determined after silencing *TcHR96h* (S3C Fig). The LC_50_ of fenpropathrin decreased in both SS and FeR strains after *TcHR96h* was silenced; however, the resistance ratio of fenpropathrin remained unchanged, further confirming that *Tc*HR96h does not play a role in fenpropathrin resistance (Table 1). By contrast, after silencing *TcHR96h*, a higher relative effect on cyflumetofen toxicity was observed in strain CyR than in SS, resulting in a dramatic decrease in the cyflumetofen resistance ratio (~44% decrease). This not only indicates that *TcHR96h* is important for cyflumetofen detoxification in both strains, but also suggests a role for *TcHR96h* in the resistance phenotype of the resistant strain (Table 1).

**Table 1:** Bioassay for the susceptible (SS), cyflumetofen-resistant (CyR) and fenpropathrin-resistant (FeR) strains after feeding on ds*TcHR96h*.

Acaricide	Lines	Treatment	χ2	LC_50_(mg/L)	95% CL	RR
Cyflumetofen	SS	ds*GFP*	4.7	2.90	2.40–3.60	-
		ds*TcHR96h*	5.0	1.50	1.20–1.80	-
	CyR	dsGFP	2.0	235	210–254	81.0
		ds*TcHR96h*	3.5	68	38–90	45.3
Fenpropathrin	SS	ds*GFP*	0.4	420	121–831	-
		ds*TcHR96h*	3.5	390	178–642	-
	FeR	ds*GFP*	0.6	70300	69900 -90300	167.3
		ds*TcHR96h*	3.2	65300	48900 -76500	167.4

Note: RR, resistance ratio = LC_50_ of ds*GFP* or ds*TcHR96h* treatment in CyR or in FeR /LC_50_ of ds*GFP* or ds*TcHR96h* treatment in SS.

### *Tc*HR96h regulates GST expression

**Fig 2:**
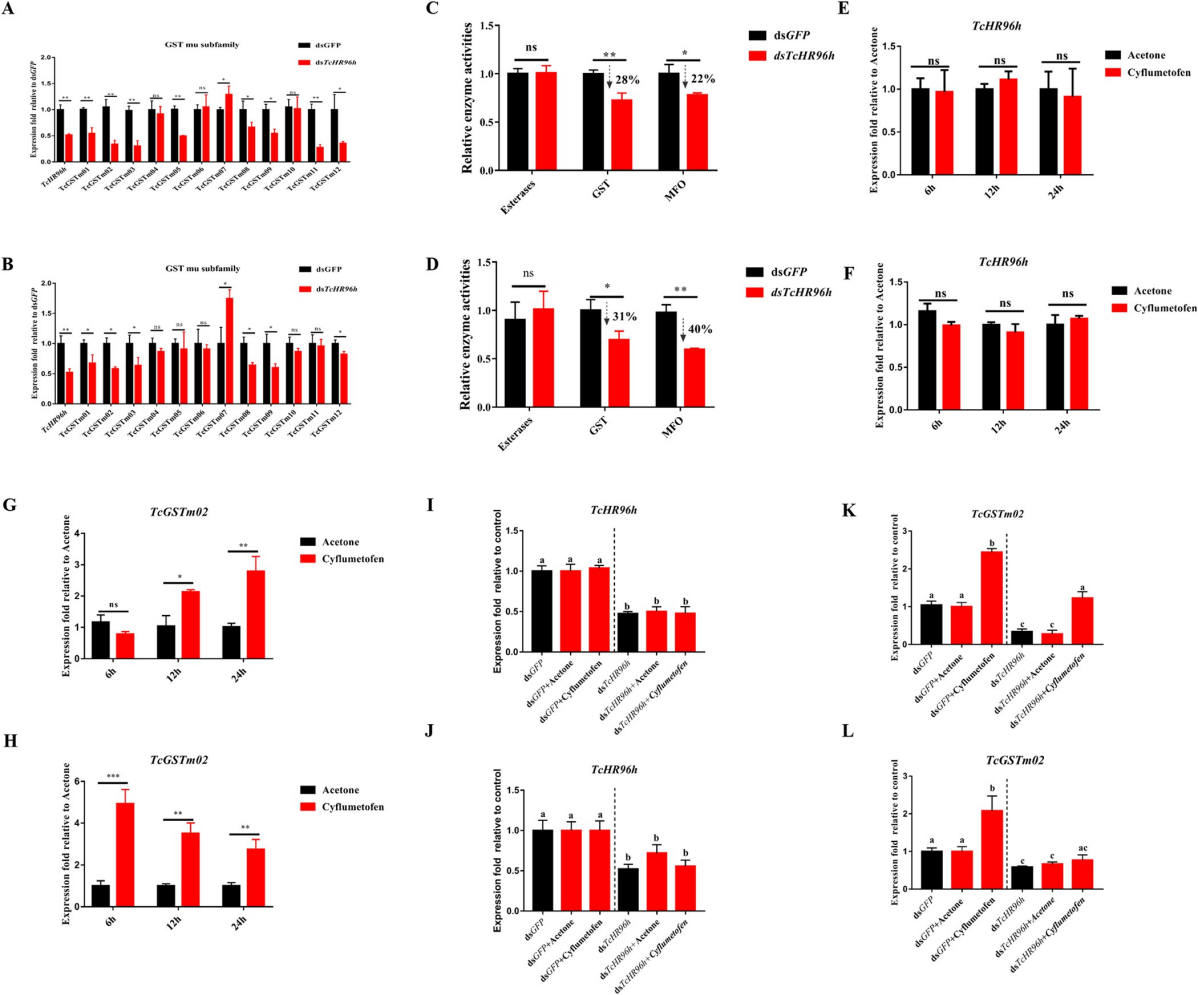
*TcHR96h* regulates GST gene expression. (A) The relative expression of GST mu subfamily genes after silencing *TcHR96h* in the susceptible strain (SS). (B) The relative expression of GST mu subfamily genes after silencing *TcHR96h* in the cyflumetofen-resistant strain (CyR). (C) The relative activities of detoxification enzymes after silencing *TcHR96h* in SS. (D) The relative activities of detoxification enzymes after silencing *TcHR96h* in CyR. The inductive expression patterns of *TcHR96h* (E) and *TcGSTm02* (G) after cyflumetofen treatment in SS. The inductive expression patterns of *TcHR96h* (F) and *TcGSTm02* (H) after cyflumetofen treatment in CyR. Cyflumetofen-induced expression of *TcHR96h* (I) and *TcGSTm02* (K) after RNAi knockdown of *TcHR96h* in SS. Cyflumetofen-induced expression of *TcHR96h* (J) and *TcGSTm02* (L) after RNAi knockdown of *TcHR96h* in CyR. (qPCR: n = 3, mean ± SE, ‘ns’ indicates no significant difference between dsTcHR96h and ds*GFP*, asterisk represents significant difference [ds*TcHR96h* compared with ds*GFP*], *P < 0.05, **P < 0.01, two-tailed Student’s *t*-test, lowercase letters indicate significant differences). Data were analyzed using ANOVA with Tukey’s HSD.

HR96 has been reported to act as a key regulator of gene expression of major detoxification gene families [17,37]. Previous work suggests that GSTs of mu subfamily play an important role in cyflumetofen resistance in this strain [35]. Therefore, the expression of 12 mu GST genes was evaluated by qPCR after silencing *TcHR96h* in SS and CyR. The expression of most genes (8/12 in SS and 6/12 in CyR) was significantly decreased (Fig 2A). In addition, the relative activity of metabolic detoxification enzymes was assessed after silencing *TcHR96h*. Compared to feeding with ds*GFP*, the relative enzyme activities of MFOs and GSTs significantly decreased by 22% (± 2%) and 28% (± 8%), respectively, after ds*TcHR96h* feeding in SS. (Fig 2C). RNAi-mediated silencing of TcHR96h in CyR, revealed a 40% (± 1%) and 31% (± 9%) reduction in the relative enzymatic activities of MFOs and GSTs, respectively, compared with ds*GFP* (Fig 2D). However, the decrease in esterase activity after ds*TcHR96h* administration in SS and CyR was insignificant.

Two GST genes were reported to be involved in the detoxification of cyflumetofen by *T*. *cinnabarinus* [35,36]. In this investigation, TcHR96h knockdown, decreased 73% (± 10%) and 42% (± 3%) of *TcGSTm02* expression in SS and CyR, respectively. However, no significant change in *TcGSTm04* expression was detected in both SS and CyR (refer to Fig 2A and 2B). To demonstrate the link between the specificity of silencing *TcHR96h* and *TcGSTm02* expression and to investigate whether *TcGSTm02* expression is induced by cyflumetofen, SS and CyR were treated with cyflumetofen before and after *TcHR96h* was silenced. When mites were directly exposed to cyflumetofen, *TcHR96h* expression was uninduced (Fig 2E and 2F). However, induction with cyflumetofen resulted in a significant upregulation of *TcGSTm02* expression (Fig 2G and 2H), suggesting that cyflumetofen treatment does not affect the expression of *TcHR96h* but triggers the induction of *TcGSTm02*. Moreover, in both SS and CyR, after significant silencing of *TcHR96h*, its expression remained stable after cyflumetofen induction, indicating that cyflumetofen exposure has no effect on *TcHR96h* expression (Fig 2I and 2J). However, when *TcHR96h* was partially silenced, *TcGSTm02* expression was less induced and failed to reach the level of the ds*GFP* control (Fig 2K and 2L), suggesting that *TcHR96h* is involved in regulating the induction of *TcGSTm02*.

### *Tc*HR96h increases transcriptional activity of *TcGSTm02* promoter

**Fig 3:**
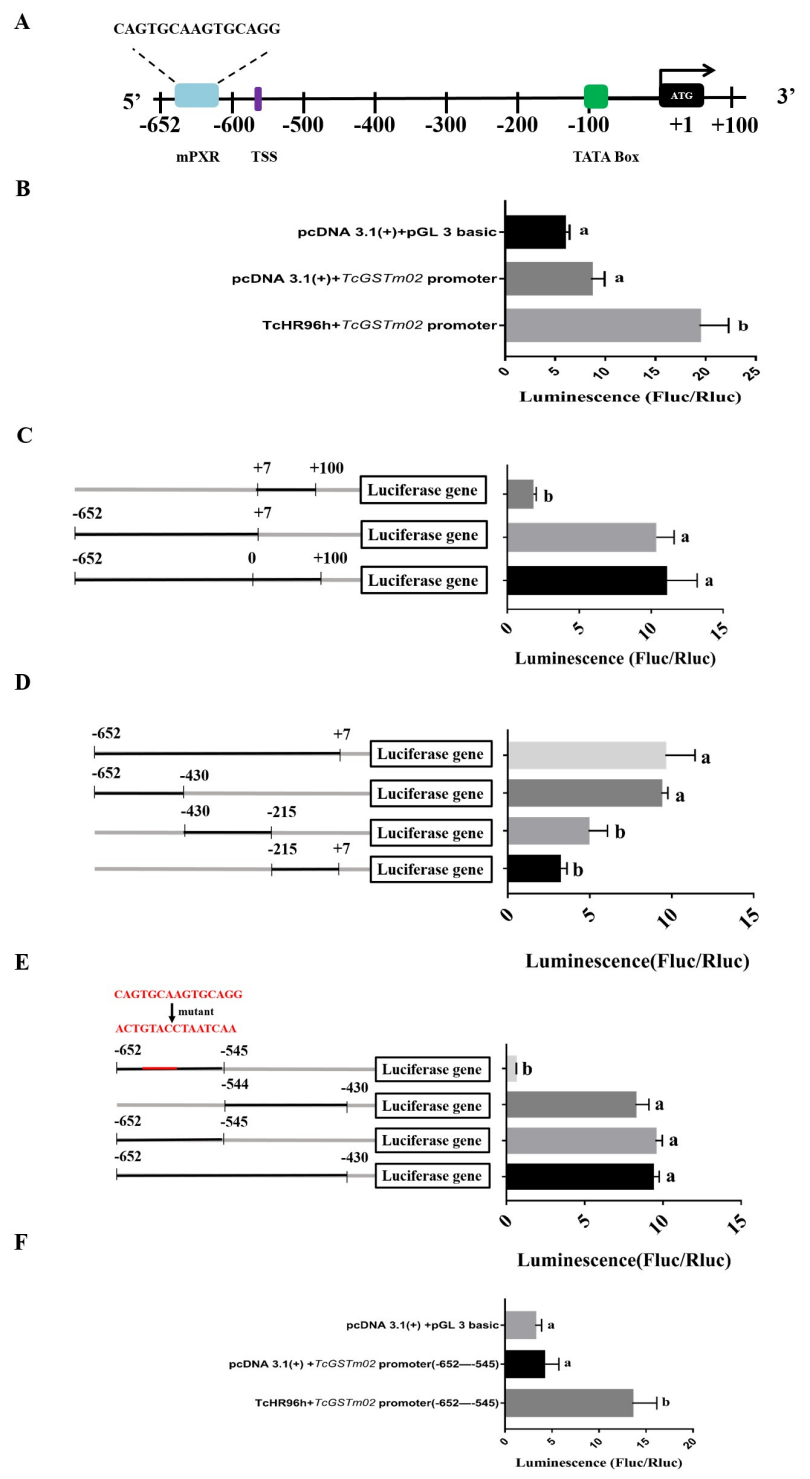
Analysis of *TcGSTm02* promoter truncations. (A) Promoter analysis of *TcGSTm02*. The green box indicates the TATA box. TSS indicates transcriptional start site. ATG indicates translation initiation region. SXR indicates the mPXR binding site predicted by ALGGEN-PROMO. Fluc/Rluc represents the ratio of firefly to Renilla luciferase activity (mean ± SE, n = 3, data were analyzed using ANOVA with Tukey’s HSD, *P* < 0.05). The solid black line represents regions that are cloned into the reporter vector. (B) Influence of *Tc*HR96h on expression driven by the *TcGSTm02* promoter in dual luciferase reporter assays. (C). The first two truncations are tested. (D). Three additional truncations are tested. (E). Two truncations of −652 to −430 fragments and a mutation truncation are tested. (F) Influence of *Tc*HR96h on expression driven by the *TcGSTm02* promoter (−652 to −545) in dual luciferase reporter assays.

To further confirm the role of *Tc*HR96h in regulating *TcGSTm02* expression, dual luciferase reporter assays were performed. The putative promoter sequence of *TcGSTm02* (Fig 3A) was initially cloned into the pGL 3.0 Basic vector, which is responsible for expressing firefly luciferase under the regulation of the cloned promoter. Subsequently, the nuclear receptor TcHR96h was cloned into pcDNA 3.1, which expresses TcHR96h under the regulation of the constitutive CMV promoter. Afterward, TcHR96h and promoter constructs were transfected into CHO cells along with pRL-TK, a control vector that expresses Renilla luciferase under the control of the constitutive HSV thymidine kinase promoter. The results showed that *Tc*HR96h increased the activity of the luciferase reporter gene by 3.29-fold compared with the control (*P* < 0.05) (Fig 3B). To further identify the *Tc*HR96h-binding sites in the *TcGSTm02* promoter, additional promoter truncation assays were conducted. The *TcGSTm02* promoter was divided into fragments, which were cloned into the pGL 3.0 Basic vector. Each construct, containing a truncation of the *TcGSTm02* promoter, was cotransfected with *Tc*HR96h constructs. After performing the dual luciferase reporter assays, the truncation that showed significantly higher (*P* < 0.05) luciferase activity was selected for generating the next set of truncations. A comparison of four truncations of the *TcGSTm02* promoter showed that the binding site might be located between −652 and +7 (Fig 3C). Three additional truncations revealed that the binding site might be located in the region between −652 and −430 (Fig 3D). Two additional truncations revealed the presence of two binding sites in fragments from −652 to −545 and −544 to −430. ALGGEN-PROMO was used to predict the nuclear receptor (NR1 family) binding motif (CAGTGCAAGTGCAGG) in the 107-nucleotide fragment located between −652 and −545 (S5 Fig). The putative binding site was further tested by introducing point mutations in a *TcGSTm02* promoter fragment (from −652 to −545). The results revealed that mutating CAGTGCAAGTGCAGG to ACTGTACCTAATCAA eliminated the reporter activity induced by *Tc*HR96h (Fig 3E and 3F). Together, these data suggest that *Tc*HR96h regulates *TcGSTm02* overexpression by binding its promoter.

### *Tc*HR96h binds cyflumetofen *in vitro*

In vertebrates, nuclear receptors bind small ligands [16,38,39]. However, whether nuclear receptors and insecticides or acaricides directly interact in arthropods is unknown. To assess the binding affinity between *Tc*HR96h and cyflumetofen and its active metabolite AB-1, molecular docking was conducted. The homology model of HR96 shows a similar overall architecture and domain organization as that of the template protein, retinoic acid receptor LXR-beta (highest similarity, 28.25%). The ligand-binding domain (LBD) comprises three α-helices and an antiparallel β-sheet. The entire ligand-binding pocket is a hydrophobic cavity that is mainly formed by hydrophobic residues from helices 3, 5, 6, 7, and 11 and two strands. Both ligands interact with two β-strands acting as a lid of the pocket through their *tert*-butyl benzene groups, and the remaining parts of the ligands are bound with a helix bundle consisting of four α-helices at the core of *Tc*HR96h (S6A and S6C Fig). The two ligands are mainly stabilized by hydrophobic interactions combined with several hydrogen bonds (S6B and S6D Fig). Cyflumetofen has an additional hydrogen bond between Arg445 and the oxygen from the branch of the molecule. Per-residue energy analysis shows that Arg448, which forms hydrogen bonds with the cyano nitrogen, contributes the most binding energy. The replacement of Arg448 with an alanine residue, with a small side chain, dramatically increases the per-residue interaction score—from −7.083 to −0.60 for cyflumetofen and −11.522 to 0.023 for AB-1.

**Fig 4:**
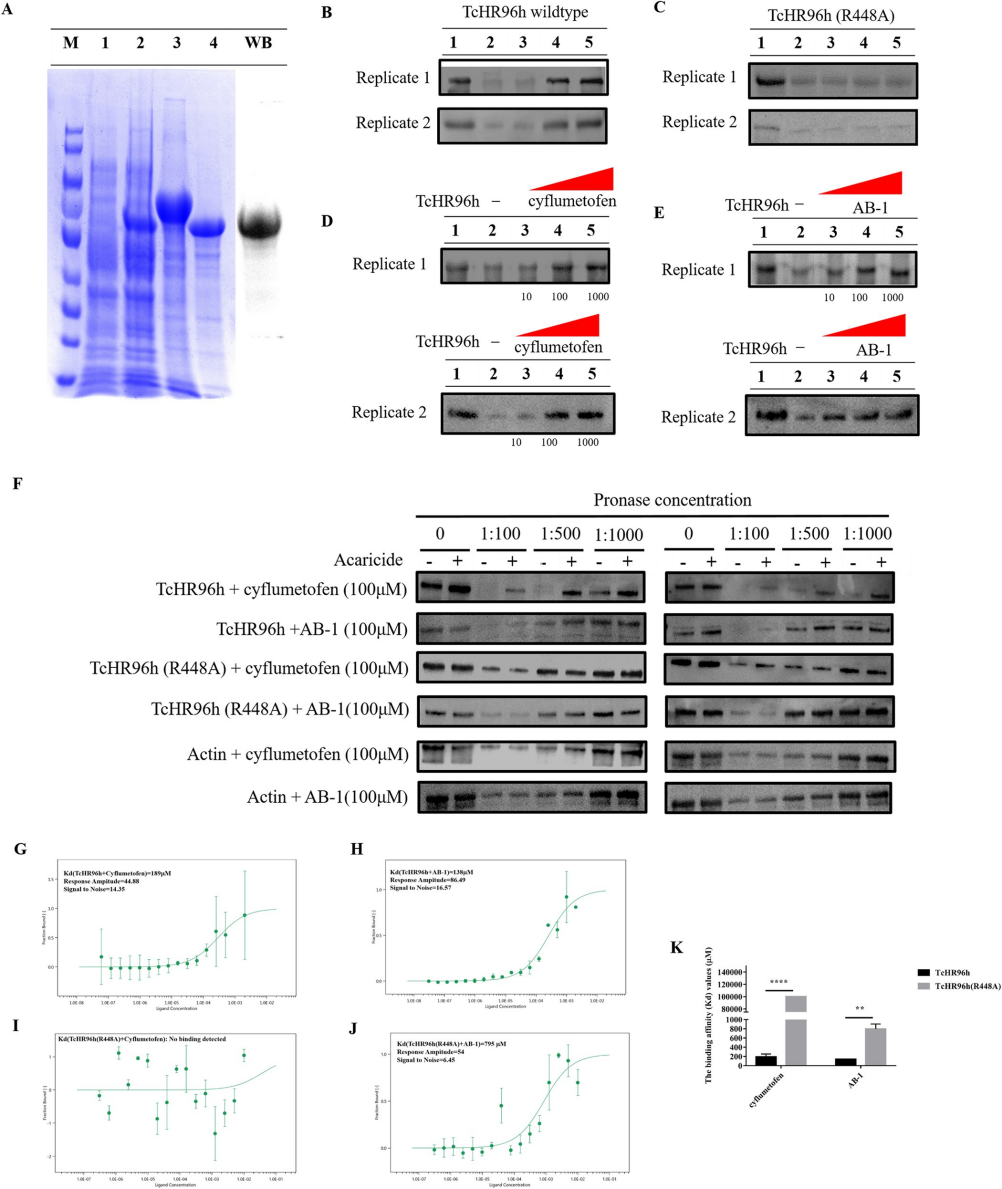
Validation of interaction between *Tc*HR96h and cyflumetofen or AB-1 using DARTs and MST. (A) SDS-PAGE analysis of recombinant *Tc*HR96h(R448A), Lane M: protein marker, Lane 1: pCold II + IPTG, Lane 2: pCold II::*Tc*HR96h (R448A) without IPTG, Lane 3: precipitated pCold II::*Tc*HR96h (R448A) + IPTG, Lane 4: soluble protein from inclusion bodies after renaturation. WB, western blotting. Interaction of *Tc*HR96h with cyflumetofen and AB-1 was measured by DARTs. (B) The affinity between *Tc*HR96h and fenpropathrin, cyflumetofen, or AB-1 was tested. The experiments were replicated two times. Lanes 1–5 represent *Tc*HR96h only, *Tc*HR96h + acetone + pronase, *Tc*HR96h + fenpropathrin + pronase, *Tc*HR96h + cyflumetofen + pronase, and *Tc*HR96h + AB-1 + pronase, respectively. (C) The affinity between *Tc*HR96h (R448A) and fenpropathrin, cyflumetofen, or AB-1 was tested. The experiments were replicated two times. Lanes 1–5 represent *Tc*HR96h (R448A) only, *Tc*HR96h (R448A) + acetone + pronase, *Tc*HR96h (R448A) + fenpropathrin + pronase, *Tc*HR96h (R448A) + cyflumetofen + pronase, and *Tc*HR96h (R448A) + AB-1 + pronase, respectively. (D) *Tc*HR96h was incubated with various concentrations (10–1000 μM) of cyflumetofen. Lanes 1–5 represent *Tc*HR96h only, *Tc*HR96h + 10 μM cyflumetofen + pronase, *Tc*HR96h + 100 μM cyflumetofen + pronase, *Tc*HR96h + 1000 μM cyflumetofen + pronase, respectively. (E) *Tc*HR96h was incubated with various concentrations (10–1000 μM) of AB-1. Lanes 1–5 represent *Tc*HR96h only, *Tc*HR96h + 10 μM AB-1 + pronase, *Tc*HR96h + 100 μM AB-1 + pronase, *Tc*HR96h + 1000 μM AB-1 + pronase, respectively. *Tc*HR96h /*Tc*HR96h (R448A) was incubated with acetone or acaricide on ice for 1 h firstly, further incubated at 37°C for 30 min and then digested with pronase (1:500) for 20 min at 37°C. *Tc*HR96h /*Tc*HR96h (R448A) without pronase was used as control. The experiments were replicated two times. (F) The DARTs experiment, employing varying concentrations of pronase along with anti-His antibodies, was conducted to investigate potential variations in protein lysis. “0” means the absence of pronase, while “1:100”, “1:500” and “1:1000” means pronase-to-protein ratio. The “-” symbol indicates the absence of acaricide, while the “+” symbol indicates acaricide added. The presence of enhanced bands (“+” lane) relative to the control conditions, where acaricide was not added (“-” lane), would indicate a binding interaction between the protein and the acaricide. The results reveal that cyflumetofen and AB-1 bind to TcHR96h against degradation, whereas TcHR96h (R448A) and actin do not. The experiments were replicated two times. Binding affinity assays of recombinant *Tc*HR96h to cyflumetofen (G), recombinant *Tc*HR96h to AB-1 (H), recombinant *Tc*HR96h (R448A) to cyflumetofen (I), and recombinant *Tc*HR96h (R448A) to AB-1 (J). MST fit curves were generated using NanoTemper analysis software 2.3.0. (K) Binding affinity (Kd) values of *Tc*HR96h and *Tc*HR96h (R448A) (mean ± SE, n = 3, two-tailed Student’s test, **P < 0.01, ****P < 0.0001).

To further confirm this interaction, recombinant *Tc*HR96h was expressed as inclusion bodies in *Escherichia coli* and ~1 mg of pure *Tc*HR96h protein was produced from 200 mL bacterial culture. Purified *Tc*HR96h and *Tc*HR96h(R448A) migrated on SDS-PAGE as a single band at ~57 kDa, which was close to the predicted molecular weight (Figs S7A and 4A). The binding ability of *Tc*HR96h and *Tc*HR96h (R448A) to acaricides was qualitatively analyzed using the DARTs strategy. The results showed that *Tc*HR96h can directly interact with cyflumetofen and AB-1 (Figs 4B and 4F, S9A and S9E) dose-dependently (Fig 4D and 4E). However, the R448A mutation dramatically reduced this binding affinity (Fig 4C and 4F). The interaction between *Tc*HR96h and cyflumetofen or AB-1 was further validated and characterized using MST. The Kd values of *Tc*HR96h binding with cyflumetofen and AB-1 were 189 and 138 μM, respectively (Fig 4F and 4G). The R448A mutation reduced the binding affinities of cyflumetofen and AB-1 by >50- and 6-fold, respectively (Fig 4H–4J).

### Cyflumetofen increases nuclear localization of *Tc*HR96h

**Fig 5:**
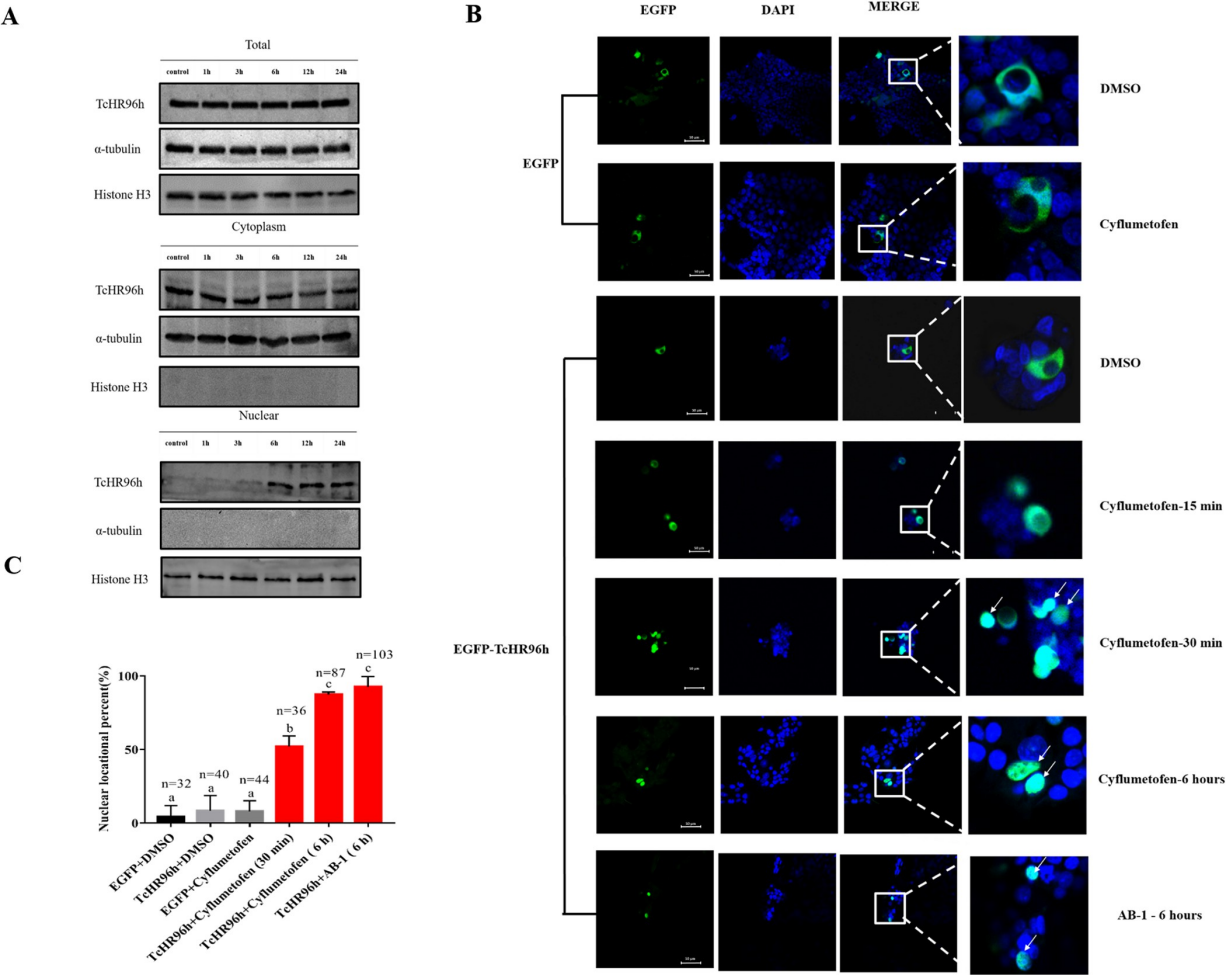
Cyflumetofen promotes translocation of *Tc*HR96h into nucleus. (A) Protein content of *Tc*HR96h in the cytoplasm and nucleus after exposure to cyflumetofen. α-Tubulin and histone H3 were used as controls in western blotting. (B) Cyflumetofen induced the nuclear translocation of EGFP-*Tc*HR96h. Scale bar, 50 μm. Arrows indicate representative areas of overlap between nuclear signal (blue, DAPI) and TcHR96h signal (green, EGFP). (C) Percentage of nuclear localization in response to cyflumetofen stimulation. The nuclear translocation ratio was calculated as the ratio of the number of cells with overlapping blue and green signals (cyan color) to the number of cells with green signals. Data were analyzed using ANOVA with Tukey’s HSD. Lowercase letters indicate significant differences (*P* < 0.05).

HR96 is expressed in the cytoplasm, but needs to be activated and translocated to the nucleus to regulate gene expression [14]. *In vitro* approaches, such as DARTs and MST, suggested that *Tc*HR96h binds cyflumetofen. Therefore, the study aimed to investigate whether *Tc*HR96h is shuttled from the cytoplasm to the nucleus after binding with and activation by cyflumetofen. To visualize this process, total protein was extracted from the cytoplasm and nucleus of mites before and after treatment with cyflumetofen. Western blot analysis revealed that in the nucleus, the content of *Tc*HR96h greatly increased after cyflumetofen stimulation over time (Fig 5A). Next, the effect of cyflumetofen on the subcellular localization of *Tc*HR96h was evaluated in HEK293T cells. The findings revealed that cyflumetofen stimulated the accumulation of EGFP-*Tc*HR96h in the nucleus. The cell culture experiments indicated that over time, following cyflumetofen treatment, the green fluorescence of EGFP-TcHR96h was transported into the nucleus, with the proportion of input to the nucleus reaching 52% and 87% after 30 min and 6 h of treatment, respectively. Similarly, the proportion of input to the nucleus reached 93% after 6 h of AB-1 treatment. (Fig 5B and 5C). Together, these data suggest that in response to cyflumetofen, *Tc*HR96h translocates to the nucleus, where it most likely binds to target DNA sequences to activate the transcription of responsive genes.

## Discussion

Phytophagous arthropods possess a powerful detoxification system not only to cope with the toxic secondary metabolites of host plants, but also to detoxify synthetic insecticides or acaricides used for their control [25,40,41]. The detoxification system in insects involves several major metabolic detoxification gene families, including cytochrome P450 [42], choline/carboxylesterase [43,44], GST [45], ABC transporter [46], and UDP-glycosyltransferase [47]. An important feature of the insect detoxification system is its ability to recruit enzymes and transporters through transcriptional upregulation upon exposure to toxicants [48]. Perturbations and genetic variations in this regulation mechanism have also been linked with the development of insecticide resistance and tolerance to plant toxins [48,49]. However, how the regulation of detoxification gene expression is activated by xenochemicals in insects is unclear. This study investigated a nuclear receptor from *T*. *cinnabarinus*- *Tc*HR96h. The findings of this study revealed that cyflumetofen directly binds to the receptor, which translocates to the nucleus and interacts with the promotor of a GST gene.

In many cases, TFs need an activation step to initiate the regulation of gene expression, and different TF families have different activation mechanisms. For example, CncC translocates to the nucleus after activation by oxidation stress [3,50]. In vertebrates, AhR is activated by recognizing and binding exogenous aromatic hydrocarbons in the cytoplasm [51,52]. However, little is known about the activation pathways of arthropod nuclear receptors, with the exception of the ecdysone receptor, which is the best-studied [14]. In this study, the conserved structure of *Tc*HR96h and the presence of a ligand binding domain suggest that *Tc*HR96h can interact with ligands, such as xenobiotics. Molecular docking between *Tc*HR96h and cyflumetofen or AB-1 indicated that *Tc*HR96h can bind cyflumetofen. The docking results propose a similar binding pose of the active metabolite of cyflumetofen, AB-1, within the binding pocket. Modeling also suggests that in *Tc*HR96h, Arg448 plays a key role in interaction with the ligand. *Tc*HR96h binding to cyflumetofen and AB-1 was confirmed using novel tools to qualities interactions between proteins and small molecules, such as DARTs [53]. The DARTs analysis reveals that TcHR96h exhibits binding affinity to cyflumetofen/AB-1 at a concentration of 100 μM. This result is further supported by the DARTs experiment conducted with different pronase concentrations. It is worth noting that the concentration of acaricide utilized in the DARTs assay was exclusively intended for in vitro observation of acaricide binding and does not reflect the actual dose encountered by mites. Finally, Kd values were calculated for the direct interaction between *Tc*HR96h and cyflumetofen or AB-1 using MST analysis. DARTs experiments showed that TcHR96h had a stronger binding affinity to cyflumetofen, while molecular docking and MST experiments indicated a stronger binding affinity to AB-1. Despite the seemingly contradictory results from different experimental methods, each method demonstrated that both cyflumetofen and AB-1 could bind effectively to TcHR96h, with binding strengths in the same order of magnitude in statistics. The relative differences in binding strengths between cyflumetofen and AB-1 at the statistical level might not have significant biological implications. By contrast, the R448A mutant of *Tc*HR96h showed dramatically reduced binding in both DARTs and MST analysis, confirming the binding model from the docking experiments. A significant increase in *Tc*HR96h was observed in the nucleus both *in vivo* and *in vitro* after exposure to cyflumetofen. These findings suggest that *Tc*HR96h, as a nuclear receptor, can be activated by binding with cyflumetofen directly and migrate to the nucleus to initiate transcription. In vertebrates, xenochemicals, such as phenobarbital, interact with both CAR and PXR; however, clotrimazole and rostanol are activators of only PXR [38]. However, in insects and mites, the direct interaction between HR96 and xenochemicals has been rarely and indirectly studied. Only one study in *Drosophila* showed that DHR96 interacts with cholesterol and regulates cholesterol homeostasis [54]. The results of this study provide a new perspective on how nuclear receptors are activated by exogenous compounds in arthropods.

How *Tc*HR96h regulates the detoxification of cyflumetofen is unclear. The data from this study show that overexpression of *TcHR96h* in CyR mites is linked with the overexpression of *TcGSTm02*. RNAi results further showed that when *TcHR96h* expression was silenced, GST activity was significantly reduced, based on a decrease in the expression of a set of GST genes. These findings suggest a pathway by which *Tc*HR96h enters the nucleus to regulate downstream genes, possibly by binding endogenous compounds, and a potential role for *Tc*HR96 in regulating the expression of GST genes without cyflumetofen exposure. Moreover, the inducible character of *TcGSTm02* under cyflumetofen exposure was confirmed with or without *TcHR96h* silencing, implying an acquired pathway of *Tc*HR96h entry the nucleus, which is driven by cyflumetofen. In addition, the luciferase assay results indicated that TcHR96h was able to regulate *TcGSTm02* promoter activity in CHO cells. In chelicerate mites, the specific response of *Tc*HR96h to cyflumetofen is an example of evolutionary safeguards against damage from xenobiotics. Furthermore, the effect was clear for *TcGSTm02*, a GST gene involved in the detoxification of cyflumetofen [35]. These results are similar to those of studies in vertebrates where GST genes are regulated by CAR and PXR [37]. Notably, DHR96 was reported to induce GST expression in *D*. *melanogaster* [7]. In *T*. *castaneum*, HR96 also plays a role in regulating the transcription of cytochrome P450 enzyme genes [55]. Thus, increased expression of *TcGSTm02* is regulated by *Tc*HR96h, which leads to increased metabolic detoxification of cyflumetofen in *T*. *cinnabarinus*.

**Fig 6:**
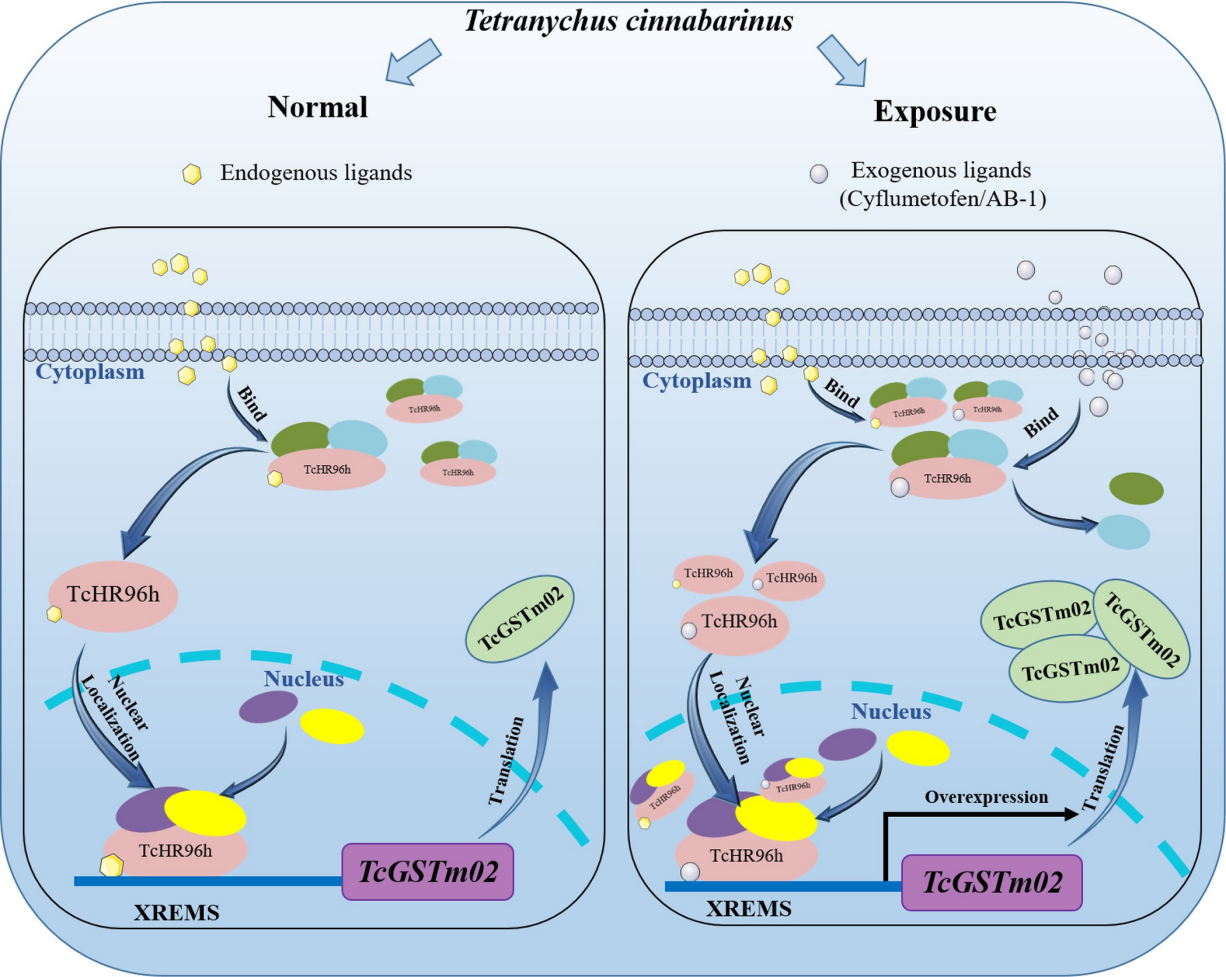
A model of *TcGSTm02* expression in response to cyflumetofen. In the proposed model, *Tc*HR96h is activated by binding the exogenous ligand (cyflumetofen/AB-1) when it enters the cytoplasm. Arginine 448 plays a key role in this binding. Once bound and activated, *Tc*HR96h localizes to the nucleus and recruits partner proteins to activate *TcGSTm02* expression by binding to the *TcGSTm02* promoter. This leads to the increased production of *TcGSTm02* and cyflumetofen detoxification.

Most organisms possess a coordinated transcriptional response to xenobiotic exposure, inducing enzymes and transporters, that facilitate detoxification [56]. The induction of detoxification genes mediated by transcriptional regulation after pesticide exposure is a crucial factor in the intrinsic toxicity of xenobiotics [4]. However, only some specific detoxification genes are activated in pests by one synthesized chemical, e.g., the expression of four P450 genes and one GST gene is upregulated by phoxim treatment in *Bombyx mori* [57]. This response to specific compounds occurs through evolutionary adaption [56]. Several TFs, like CnCC/Keap1, Maf, and nuclear receptor, have been identified in vertebrates that contribute to this regulatory response [48]. By contrast, little is known about the initiation of this pathway in invertebrates and whether TFs directly interact with xenobiotics. To our knowledge, this study is the first to report that nuclear receptors can directly bind with the toxicant and initiate detoxification gene expression to ensure xenobiotic detoxification. In the proposed model (Fig 6), *Tc*HR96h is activated by binding cyflumetofen when it enters the cytoplasm, with a key role played by Arg448. Once bound and activated, *Tc*HR96h localizes to the nucleus and recruits partner proteins to activate *TcGSTm02* expression by binding to the promoter of this gene, which leads to increased *Tc*GSTm02 production and cyflumetofen detoxification. Although qPCR experiments are routinely performed to confirm differentially expressed genes based on RNAseq data, a discrepancy between qPCR and RNAseq data has often been observed [58]. In our results, although there seems discrepancy between RNA-seq and qPCR data, both methods show a confirmed over-expression of TcHR96h. In strains of CyR and YN-CyR, an elevated amount of TcHR96h could be driven into the nucleus more effectively under cyflumetofen exposure. This enhanced translocation is a result of the increased binding cyflumetofen molecules due to the overexpression of TcHR96h. Consequently, the transcription and detoxification process in the CyR and YN-CyR populations could be significantly enhanced. In our previously published studies, we have shown that non-coding RNAs exert post-transcriptional regulatory control over the upregulation of *TcGSTm02* [59]. The combined effect of transcriptional and post-transcriptional regulation contributes to the overexpression of *TcGSTm02*, which in turn enhances the detoxification ability of CyR strains.

Specific response to artificial chemicals could be an adaptive strategy in arthropods and might be linked with the early adaptation to host allelochemicals. Studies suggest that generality in diet breadth was achieved by disrupting the defense signals or acquire the detoxification strategies of the host plant by horizontal gene transfer. In *Bemisia tabaci*, *BtPMaT1*, which was acquired from the host plant, allowed whiteflies to neutralize phenolic glucosides and broaden the spectrum of defensive compounds [60]. In *T*. *urticae*, 17 intradiol ring-cleavage dioxygenases-a set of genes transferred horizontally-play a role in the xenobiotic response [22]. Horizontal gene transfer may have provoked the nonspecific adaptive evolution of arthropods. The interaction between nuclear receptors and xenobiotics may be another strategy to obtain optimal diet breadth for spider mites, which also have a wide range of hosts. Due to the expansion of the HR96 family, mites can recognize a wide range of plant allelochemicals through direct binding and then adapting to and feeding on a wider range of host plants. Although this study has only examined *Tc*HR96h in detail, the nonspecific adaptive response is worthy of further exploration. Specific and general adaptive mechanisms might be a combined strategy for polyphagous herbivores to adapt to plant alleochemicals and artificial pesticides.

In summary, we found that specifically *TcHR96h* was 18.2-fold higher expressed in the cyflumetofen-resistant strain. Moreover, the down-regulation of *TcHR96h* via RNAi confirmed its key role in mediating cyflumetofen detoxification. We also show that *TcHR96h* can be activated by cyflumetofen through direct binding, after which it translocates to the nucleus where it regulates the expression of *TcGSTm02* via promotor interactions.

## Materials and methods

### Mite strains

The susceptible strain (SS), collected in 1998, from Beibei District, Chongqing, China, was maintained on fresh cowpea (*Vigna sinensis*) leaves for >20 years without exposure to pesticides [61]. Strain CyR (resistance ratio [RR] ~100-fold)—a substrain originating from the SS strain—was selected using cyflumetofen in the laboratory [33]. The fenpropathrin-resistant (FeR) strain (resistance ratio [RR] >100-fold)—a substrain originating from the SS strain—was selected using fenpropathrin in the laboratory [62]. Strains SS, CyR, and FeR were maintained in incubators at 26°C ± 1°C and 55%–75% relative humidity (RH), with a photoperiod of 14:10 h (L: D).

### Extraction of DNA and RNA, cDNA synthesis, and RT-qPCR analysis

Genomic DNA was extracted from ~200 female adult mites (3–5-d-old) of the SS strain using DNA Extraction Reagent (Solarbio, Beijing, China). Total RNA was isolated from ~200 female adult mites (3–5-d-old) using TRIzol reagent (Thermo Fisher Scientific Inc., USA). RNA quality was analyzed by 1% agarose gel electrophoresis and a NanoDrop 2000 spectrophotometer (Thermo Fisher Scientific Inc., USA) and genomic DNA was removed from total RNA using DNase. First-strand cDNA was synthesized using PrimeScript 1st Strand cDNA Synthesis Kit (Takara Biotechnology Dalian Co., Ltd., Dalian, China) and stored at −20°C.

Quantitative PCR (qPCR) primers were designed by Primer Premier 5.0 (PREMIER Biosoft International, Palo Alto, California, USA), and ribosomal protein S18 (RP18S, FJ608659) and tubulin alpha-I chain (α-Tub, FJ526336) were used as reference genes (S1 Table). All primers were first tested for a gradient concentration template using the qPCR system. Primers with high amplification efficiency (90%–110%) and a single peak in melting curve analysis were selected. The reaction system consisted of 1 μL of diluted cDNA, 10 μL of GoTaq qPCR Master Mix, 7 μL of nuclease-free water, and 0.2 mM of each primer. Thermal cycling conditions were as follows: 95°C for 2 min, 95°C for 15 s, 60°C for 30 s, and melting curve analysis from 60°C to 95°C; steps 2–4 were repeated 40 times. Three biological replicates and three-technique replicates were used. The 2^-ΔΔCt^ method was used to analyze expression level, significant differences (*P* < 0.05) were evaluated using SPSS 22.0 (SPSS Inc., Chicago, IL, USA) for Windows [63].

### Molecular cloning and bioinformatics analysis

TcHR96h sequence information was acquired from NCBI, as described [64]. The open reading frames were cloned from the SS, CyR, and FeR strain, respectively and nucleotide sequence alignment was performed (S2A Fig). The conserved domains were detected using bioinformatics tools of the NCBI server (S2B Fig). Theoretical isoelectric point (pI) and molecular weight (MW) were computed using the Compute pI/Mw tool (https://web.expasy.org/compute_pi/). Detailed ORF information for TcHR96h is shown in S2 Table. The phylogenetic trees, based on amino acid sequence alignment, were constructed using MEGA 7.0 (maximum likelihood LG + G method) and boot-strapped with 1000 replicates (S2C Fig). The amino acid sequences of genes, identified in different insect species, were downloaded from the NCBI database (S3 Table).

### dsRNA feeding

Partial sequences of TcHR96h and the green fluorescent protein (GFP) gene (NCBI: ACY56286, negative control, sequence fragment: 435 bp) were obtained by PCR with specific primers containing T7 RNA polymerase promoter (S1 Table). The dsRNAs were synthesized using Transcript Aid T7 High Yield Transcription Kit (Thermo Scientific, Vilnius, Lithuania). The dsRNAs were suspended in nuclease-free water at ~1000 ng/μL and stored at −80°C. The leaf-disc dsRNA feeding method was used to deliver dsRNA. After the dsRNA solution was completely absorbed by square cowpea leaves (4 cm^2^), which had been slightly dehydrated at 60°C for 2 min, the leaves were placed on wet filter paper. Each leaf disc was shared by 80 female adults (3–5-d-old and starved for 24 h) for 72 h feeding under controlled conditions (26 ± 1°C, 55% relative humidity and 14:10-h light/dark photoperiod). Mites were then harvested for RT-qPCR, bioassays, insecticide exposure test, and enzyme activity assay. Three biological replicates were used for the experiments.

To test RNAi efficiency, the relative expression of TcHR96h was quantified by comparing mRNA levels in mites fed with dsRNAs of GFP or TcHR96h. dsRNAGFP was used as the control, and silencing efficiency was expressed as percentage.

At 72 h after dsRNA feeding, samples were also collected for the bioassays, insecticide exposure test, enzyme assays, and qPCR analysis. The activity levels of MFO, esterase, and GST were determined. The expression levels of GST mu genes, after 72 h of dsRNA feeding, were determined through qPCR.

### Bioassay and insecticide exposure

The residual coated vial method was used for the cyflumetofen bioassay [65] and the leaf-disc spraying method for the fenpropathrin bioassay [66]. Cyflumetofen was dissolved in acetone and diluted to an appropriate concentration range. Fenpropathrin was first dissolved in acetone to make a stock solution and then diluted to an appropriate concentration range in deionized water. Each concentration was assessed in replicates. LC_50_ value and 95% confidence limit (CL) were calculated using SPSS 22.0 and probit analysis.

For exposure experiments, cyflumetofen was dissolved in acetone. Then, 30% lethal concentration (LC_30_) values of cyflumetofen were calculated, and carmine spider mites were treated with the LC_30_ concentration of cyflumetofen using the residual coated vial method. Samples were collected at 6, 12, and 24 h after exposure. The acetone-only treatment group was used as a control. Each insecticide treatment group contained ~200 mites, and three biological replicates were performed.

### Enzyme assays

SS and CyR strains were used to determine the activity of detoxification enzyme. Total protein content of the enzyme solution was determined by the Bradford method [67], with bovine serum albumin (BSA) as the standard.

Mixed function oxidase (MFO) activity was tested according to Shang’s method [68]. Briefly, 200 female adult mites were homogenized in 1 mL PBS (0.2 mol/L, pH 7.8) on ice. The homogenate was centrifuged at 10,000 × *g* for 15 min at 4°C, and the supernatant was used as enzyme source. The enzyme solution and NADPH were added to the substrate *p*-nitroanisole (0.1 mol/L in acetone) and incubated for 30 min at 37°C. The reaction was stopped by adding 1 mol/L hydrochloric acid, extracted with chloroform and 0.5 mol/L NaOH, and read at 400 nm using a microplate reader (EON, BioTek Instruments Inc., Winooski, Vermont, USA). Specific activity was calculated based on a nitrophenol standard curve and protein concentration of enzyme source. Three independent crude enzyme extracts, representing three biological replicates, were prepared and analyzed. Results are expressed as mean activity (± SE, standard error).

Glutathione-S-transferase (GST) activity was determined as described [69]. Briefly, 200 female adult mites were homogenized in 1 mL PBS (0.04 mol/L; pH 7.5) on ice, followed by centrifugation at 10,000 × *g* for 10 min at 4°C. CDNB (0.6 mmol/L) and GSH (6 mol/L) were used as substrates for the enzyme assay. First, 100 μL CDNB and 100 μL GSH were incubated at 37°C for 20 min, and then, 100 μL enzyme was added to the reaction system. Optical density at 340 nm was immediately recorded at intervals of 30 s for 5 min using a microplate reader (EON, BioTek Instruments Inc., Winooski, Vermont, USA). Three independent crude enzyme extracts, representing three biological replicates, were analyzed. Results are expressed as mean activity (± SE, standard error).

The method reported by Vanasperen was adopted for testing esterase activity [70]. Briefly, 200 female adult mites were homogenized in 1 mL PBS (0.04 mol/L; pH 7.5) on ice, followed by centrifugation at 10,000 × *g* for 10 min at 4°C. The supernatant was kept on ice until testing. Using α-naphthyl acetate (3 × 10^−4^ mol/L) as substrate for acetylcholinesterase activity, the reaction mixture was incubated for 10 min at 37°C, after which the color developing agent (5% SDS:1% Fast Blue B Salt = 5:2, v/v) was added and the OD value was immediately recorded at 600 nm using a microplate reader (EON, BioTek Instruments Inc., Winooski, Vermont, USA). The specific activity of esterase was calculated based on an α-naphthol standard curve and protein concentration of enzyme source. Three independent crude enzyme extracts, representing three biological replicates, were analyzed. Results are expressed as mean activity (± SE, standard error). Standard curves for all assays are shown in S4 Fig.

### Cell culture and vector construction

Chinese hamster ovary (CHO) and HEK293T cells were maintained at 37°C under 5% CO_2_ in DMEM/F-12 medium (Gibco BRL, Gaithersburg, MD, USA) with 10% fetal bovine serum (FBS) (Gibco, USA) and antibiotics. Promoter sequence information for *TcGSTm02* was obtained from the genome database of *T*. *urticae*. Promoter regions were amplified using genomic DNA extracted from female adults (3–5-d-old) of *T*. *cinnabarinus*. The primers used for *TcGSTm02* promoter is shown in S1 Table. The TATA box and transcription start site were predicted and analyzed using an online software (http://www.fruitfly.org and http://www.softberry.com). Potential binding motifs of *Tc*HR96h were predicted using the TF number T05236, T05257, T05670, and T05671 in the ALGGEN-PROMO database [71,72]. The promoter region and various DNA fragments for *TcGSTm02* promoter truncations and mutated promoter truncations were artificially synthesized into pGL 3.0 Basic using the *TcGSTm02* promoter DNA as a template (TsingKe Biological Technology Co., Ltd. (TsingKe, China). The *Tc*HR96h ORF was cloned into the expression vector pcDNA3.1 and inserted into the overexpression vector pEGFP.

### Dual-luciferase reporter assay

Transfection was performed in 6-well cell culture plates and promoter analysis was performed using CHO cells. A total of 100 μL of cells (1 × 10^6^ cells/mL) were pipetted into a single well and incubated in DMEM/F-12 medium (10% FBS with 1% penicillin and 1% streptomycin) at 37°C and 5% CO2 for 24 h. To evaluate TcHR96h regulatory activity, the following transfection groups were made: 1.5 μg pcDNA 3.1 + 1.5 μg pGL 3.0 Basic + 150 ng pRL-TK, 1.5 μg pcDNA 3.1 + 1.5 μg pGL 3.0 basic::TcGSTm02 promoter or 1.5 μg pGL 3.0 Basic::TcGSTm02 promoter (−652 to −545) + 150 ng pRL-TK and 1.5 μg pcDNA 3.1::TcHR96h + 1.5 μg pGL 3.0 Basic::TcGSTm02 promoter or 1.5 μg pGL 3.0 Basic::TcGSTm02 promoter (−652 to −545) + 150 ng pRL-TK. The pGL 3.0 basic reporter plasmids carrying the indicated promoter regions conjugated to firefly luciferase and a reference reporter plasmid (pRL-TK, containing the Rluc reporter gene and an HSV TK promoter) were used to transfect cells with 8 μL of Attractene Transfection Reagent (QIAGEN, Germany) in 500 μL of DMEM/F-12 medium without FBS and antibiotics. First, two promoter truncation analyses were performed with the following transfection groups: 1.5 μg pcDNA 3.1::TcHR96h + 1.5 μg pGL 3.0 Basic::TcGSTm02 promoter + 150 ng pRL-TK, 1.5 μg pcDNA 3.1::TcHR96h + 1.5 μg pGL 3.0 Basic::TcGSTm02 promoter (−652 to +7) + 150 ng pRL-TK and 1.5 μg pcDNA 3.1::TcHR96h + 1.5 μg pGL 3.0 Basic::TcGSTm02 promoter (+7 to +400) + 150 ng pRL-TK. Second, three promoter truncation analyses were performed with the following transfection groups: 1.5 μg pcDNA 3.1::TcHR96h + 1.5 μg pGL 3.0 Basic::TcGSTm02 promoter (−652 to +7) + 150 ng pRL-TK, 1.5 μg pcDNA3.1::TcHR96h + 1.5 μg pGL 3.0 Basic::TcGSTm02 promoter (−652 to −430) + 150 ng pRL-TK, 1.5 μg pcDNA 3.1::TcHR96h + 1.5 μg pGL 3.0 Basic::TcGSTm02 promoter (−430 to −215) + 150 ng pRL-TK and 1.5 μg pcDNA3.1::TcHR96h + 1.5 μg pGL 3.0 Basic::TcGSTm02 promoter (−215 to +7) + 150 ng pRL-TK. Third, three promoter truncation analyses were performed with the following transfection groups: 1.5 μg pcDNA 3.1::TcHR96h + 1.5 μg pGL 3.0 Basic::TcGSTm02 promoter (−652 to −430) + 150 ng pRL-TK, pcDNA 3.1::TcHR96h + 1.5 μg pGL 3.0 Basic::TcGSTm02 promoter (−652 to −545) + 150 ng pRL-TK, 1.5 μg pcDNA 3.1::TcHR96h + 1.5 μg pGL 3.0 Basic::TcGSTm02 promoter (−544 to −430) + 150 ng pRL-TK and 1.5 μg pcDNA 3.1::TcHR96h + 1.5 μg pGL 3.0 Basic::TcGSTm02 promoter (−652 to −545, mutant) + 150 ng pRL-TK. The samples were incubated for 6 h. Then, DMEM/F-12 medium was removed and 2 mL of fresh DMEM/F-12 medium, with 10% FBS and antibiotics, was added to each well. The cells were harvested by centrifugation at 10,000 × *g* for 5 min after 48 h of transfection. Then, dual-luciferase activity was measured using Dual Luciferase Assay System E2920 (Promega, Madison, WI, USA). Results are expressed as the ratio of firefly luciferase activity/Renilla luciferase activity. Three biological replicates in each setup for each transfection group and three technical replicates for luciferase activity assay were used.

### *Tc*HR96h and actin expression and purification

The procedures for protein expression were as described [35]. The primers used are shown in S1 Table. Briefly, the coding region of TcHR96h, TcHR96h (R448A) or actin was cloned into the pCold II vector. For protein production, the recombinant vector was transformed into Rosetta-gami B(DE3) competent cells. A single colony was cultured at 37°C in LB-ampicillin medium, supplemented with 100 mg/mL ampicillin until OD600 reached 0.6–0.8. The culture was induced with 0.5 mM IPTG, cultured for 12 h at 15°C, and harvested by centrifugation at 4000 × *g* for 30 min. The harvested cells were disrupted using sonication in Buffer I (0.04 M PBS; pH 7.4) on ice. The cell supernatant was removed by centrifugation for 20 min at 10,000 × *g*. The pellet was washed with Buffer II (100 mM PBS, 10 mM EDTA, 100 mM NaCl; pH 8.0) and Buffer III (50 mM PBS, 2 M urea; pH 8.0); denatured with Buffer IV (100 mM PBS, 8 M urea, 0.2 M DTT; pH 8.0); and dialyzed and concentrated after overnight renaturation with Buffer V (100 mM PBS, 1 mM EDTA, 0.9 mM GSH, 0.18 mM GSSH, and 2 M urea; pH 8.0). Finally, the purified protein was obtained from inclusion bodies.

### Molecular docking, DARTs, and MST

The homology model of full-length HR96 was built using the Prime module in Schrödinger. The crystal structure of the retinoic acid receptor LXR-beta was used as template (PDB ID: 4NQA). The R448A mutation was introduced into the built homological model using UCSF chimera, followed by energy minimization with Amberff99 force field. Two ligands, cyflumetofen and AB-1, were drawn using ChemDraw and prepared using the LigPrep module in Schrodinger. The two-dimensional structures were converted into energy-minimized three-dimensional structures, using OPSL3 force field, at neutral pH [73]. The two ligands were docked into homological models of HR96 using Glide XP (Schrödinger LLC, New York, NY, USA) [74]. The key residues around the hypothetical binding pocket were called the receptor box center, and ligands were docked within 20 Å. A maximum of 10 poses were generated for each ligand. Docking scores, reflecting relative binding affinities of ligands, were used to rank-order the ligands.

Drug affinity responsive target stability (DARTs) was performed as described, with some optimization [75]. DARTs leverages the thermodynamic stabilization of the target protein that occurs upon small-molecule binding by detecting the binding-induced increase in resistance to proteolysis. In other words, the stronger the protein’s ability to bind small molecules, the less the protein–small molecule complex is degraded by proteases, and the more visible the bands are in western blot analysis. In this study, 20 μg TcHR96h was gently mixed with fenpropathrin, cyflumetofen (10–1000 μM), or AB-1 (10–1000 μM). The samples were first incubated on ice for 1 h and then at 37°C for 30 min. TcHR96h without pronase and TcHR96h + acetone were used as controls. After incubation, the samples were treated with 40 ng pronase (33 nM, mass ratio between pronase and protein, 1:500) for 20 min at 37°C. The reaction was terminated with a protease inhibitor and the samples were analyzed using SDS-PAGE and western blotting. DARTs were also performed at different concentrations of pronase (mass ratio between pronase and protein 1:100, 1:500 and 1:1000, resulting in molar concentrations of 167 nM, 33 nM and 17 nM, respectively.). Two independent replicates were analyzed. The relative band intensity was expressed in gray values using ImageJ. The relative gray value for the TcHR96h + acetone control group was set to one; normalization was done per gel.

The fluorescence of the purified protein bound to the insecticides was evaluated using microscale thermophoresis technology (MST), according to the manufacturer’s protocol. Monolith Protein His-Tag Labelling Kit RED Tris-NTA 2nd Generation (Nano Temper Technologies GmbH, Munich, Germany) was used to label purified TcHR96h and TcHR96h (R448A). Labeling was performed according to the manufacturer’s instructions. Briefly, based on the affinity of the dye to the His-tagged protein, the labeled protein and dye were diluted to 4 μM and 50 nM, respectively, using 1× PBS with 0.05% Tween 20 (PBST) and mixed (1:1) at 25°C for 30 min. The insecticide ligands were dissolved in DMSO. A series of 16 dilutions (1:1) of the ligand was prepared using PBST. The first concentration of cyflumetofen and AB-1 was 2 mM (2% DMSO) for TcHR96h and 10 mM (2% DMSO) for TcHR96h (R448A). An equal volume of labeled protein was added to each of the 16 concentrations of the ligands and mixed well. The samples were loaded and measured at 40% LED/excitation power and medium MST power. System default values were used for the other parameters. Two or three independent replicates were analyzed to evaluate the binding affinity (KD value) using the MO Affinity Analysis software (version 2.3.0).

### Protein extraction from spider mites and western blotting

Adult female mites were sprayed with a sublethal concentration of cyflumetofen solution, and surviving female mites were collected after 1, 3, 6, 12 and 24h exposure at approximately 40 mg for protein extraction [76]. Total protein was extracted from *T*. *cinnabarinus* using Minute Total Protein Extraction Kit for Insects (Invent). Total cytoplasmic protein and total nuclear protein were extracted from *T*. *cinnabarinus* using Nuclear Protein Extraction Kit (Solarbio, Beijing, China), strictly following the manufacturer’s protocol. α-Tubulin and histone H3 were used as controls in western blotting.

To ensure antibody specificity, similarity analysis between TcHR96h and eight TuHR96 genes was conducted (S4 Table). A rabbit polyclonal antibody against recombinant TcHR96h was generated by GeneCreate Biological Engineering Co., Ltd. (GeneCreate, China) and diluted with Tris-buffered saline–Tween solution at a ratio of 1:8000 before use. Proteins were first separated by 10% SDS-PAGE, and subsequently, transferred to 0.45-μm polyvinylidene fluoride membranes. Membranes were blocked with 5% skim milk for 1 h and incubated with the TcHR96h antibodies overnight at 4°C. Subsequently, the membranes were washed and incubated with goat anti-rabbit Immunoglobulin G (dilution rate 1: 20,000) (CwBio, Beijing) for 1 h. Finally, protein bands were analyzed using enhanced chemiluminescence (ECL) (Bio-Rad, USA). Exposure time for TcHR96h in the nuclear fraction was 90 s and that for the rest was 20 s. α-Tubulin (AF0001, Beyotime, China) and histone H3 (AH433, Beyotime, China) were used as controls in western blotting and diluted with Tris-buffered saline-Tween solution at a ratio of 1:5000 (α-tubulin) and 1:1000 (histone H3) before use.

### *Tc*HR96h subcellular location in HEK293T

See section of Cell culture and vector construction for the construction of the pEGFP and pEGFP::TcHR96h vectors. HEK293T cells were chosen for transfection with pEGFP and pEGFP::TcHR96h using Attractene Transfection Reagent. The cells were examined 48 h after transfection using a laser confocal microscope for green fluorescence to examine vector expression. The cells were subsequently treated with DMSO as a control or with 200 μM cyflumetofen. After 15 or 30 min, the cells were washed three times with DMEM/F-12 medium and the nuclei were labeled with DAPI. The cells were then analyzed and fluorescence images were acquired using the Zeiss LSM 780 confocal microscope (Carl Zeiss SAS, Germany). Positive results were identified when green fluorescence overlapped with blue DAPI signal. One representative view in a well was selected for cell counting. Each treatment group had three biological replicates (3 wells). In a well, the number of cells expressing green fluorescence (total) and cells with overlapping blue and green signals after cyflumetofen/AB-1 treatment (positive) was recorded individually. Raw data of cell counts are presented in S5 Table. The nuclear translocation ratio was calculated as the ratio of the number of cells with overlapping blue and green signals (cyan color) to the number of cells with green signals.

### Statistical analysis

The statistical significance of differences between samples was analyzed using Student’s *t*-test and ANOVA with Tukey’s HSD. All quantitative data are reported as means ± standard error (SE) from three independent experiments. SPSS 22.0 statistical software was used in this study.

## Supporting information

S1 Fig HR96 expression level of strains CyR and YN-CyR. Canonical HR96 (HR96a-h) is marked in the heatplot and the other genes are HR96-like genes. Asterisk represents significant difference (CyR compared with SS or YN-CyR-L/M/H compared with YN-S), |log_2_ ratio| ≥ 1. The cyflumetofen-resistant strain (YN-CyR) was selected from YN-S which was collected from fields in Yunnan, China by continuous selection with cyflumetofen. The resistance ratio of YN-CyR_L, YN-CyR_M, and YN-CyR_H reached 7.83-, 17.23-, and 86.05-fold, respectively and which were named low (L), medium (M), and high levels of resistance (H).
(TIF)

S2 Fig Identification and sequence analysis of *TcHR96h*. (A) *TcHR96h* nucleotide sequence comparisons in SS, CyR and FeR strains. (B) Conserved sequence analysis of *Tc*HR96h. NR_DBD indicates DNA binding domain and NR_LBD indicates ligand binding domain. (C) Phylogenetic analyses of *TcHR96h*. The phylogenetic trees were constructed for amino acid sequences by the maximum likelihood LG + G method. Bootstrap analyses were performed with 1000 iterations, and only bootstrapping values >50 are shown.
(TIF)

S3 Fig Relative expression of *TcHR96h* and *TcHR96d* mRNA after *TcHR96h* RNAi. (A) The design of the qPCR and dsRNA fragments. (B) Relative expression of *TcHR96h* mRNA after RNAi in strains SS and CyR was assessed by qPCR (qPCR: n = 3, mean ± SE, asterisk represents significant difference (ds*TcHR96h* compared with ds*GFP*), *P < 0.05, **P < 0.01, two-tailed Student’s *t*-test). (C) Relative expression of *TcHR96h* mRNA after RNAi in strains SS and FeR was assessed by qPCR (qPCR: n = 3, mean ± SE, asterisk represents significant difference (ds*TcHR96h* compared with ds*GFP*), *P < 0.05, **P < 0.01, two-tailed Student’s *t*-test). ds*GFP*, the dsRNA of green fluorescent protein, was used as a negative control. ds*TcHR96h*, the dsRNA of *TcHR96h*. Downward arrows indicate decreased expression of *TcHR96h*, and silencing efficiency is expressed as percentage. (D) Relative expression of *TcHR96d* mRNA after RNAi in strains SS and CyR was assessed by qPCR (qPCR: n = 3, mean ± SE, ‘ns’ indicates no significant difference between ds*TcHR96h* and ds*GFP*, two-tailed Student’s *t*-test). (E) Relative expression of *TcHR96d* mRNA after RNAi in strains SS and FeR was assessed by qPCR (qPCR: n = 3, mean ± SE, ‘ns’ indicates no significant difference between ds*TcHR96h* and ds*GFP*, two-tailed Student’s *t*-test). *TcHR96d* was used to the analyze off-target effects of *TcHR96h* RNAi.
(TIF)

S4 Fig Standard curves used in enzyme assays. Standard curves of (A) BSA, (B) *p*-nitrophenol, and (C) of α-naphthol.
(TIF)

S5 Fig Output of online software ALGGEN-PROMO for predicting binding motif of NR1 family at *TcGSTm02* promoter. (A) *TcGSTm02* promoter truncation from −652 to −545. Only one motif has been predicated. (B) *TcGSTm02* promoter truncation from −544 to −430. No motif has been predicated.
(TIF)

S6 Fig Docking models of cyflumetofen and AB-1 with *Tc*HR96h. (A) The binding pose of cyflumetofen (green) with *Tc*HR96h. (B) The ligand interaction diagram of cyflumetofen. (C) The binding pose of AB-1 (purple) with *Tc*HR96h. (D) The ligand interaction diagram of AB-1. The side chains of ligand-coordinating residues are displayed and labeled. Hydrogen bonds are indicated using orange dashed lines.
(TIF)

S7 Fig SDS-PAGE analysis of recombinant *Tc*HR96h and actin. (A) SDS-PAGE analysis of recombinant *Tc*HR96h. Lane M: protein marker, Lane 1: pCold II + IPTG, Lane 2: pCold II::*Tc*HR96h without IPTG, Lane 3: pCold II::*Tc*HR96h + IPTG, Lane 4: supernatant of pCold II::*Tc*HR96h + IPTG, Lane 5: precipitate pCold II::*Tc*HR96h + IPTG, Lane 6: Inclusion bodies denaturing solution, Lane 7: soluble protein Ι from inclusion bodies after renaturation, Lane 8: soluble protein II from inclusion bodies after renaturation, WB: western blotting. (B) Nucleotide sequence alignment between *Tc*HR96h and *Tc*HR96h (R448A). Mutation from G to C at position 1346. (C) SDS-PAGE analysis of recombinant actin. Lane M: protein marker, Lane 1: pCold II + IPTG, Lane 2: pCold II::*actin* without IPTG, Lane 3: pCold II::*actin* + IPTG, Lane 4: precipitates of pCold II::*actin* + IPTG, Lane 5: upernatant pCold II::*actin* + IPTG, Lane 6: soluble actin protein from inclusion bodies after renaturation, Lane 7: western blotting.
(TIF)

S8 Fig DARTs experiment using varying concentrations of pronase. The DARTs experiment, employing varying concentrations of pronase along with anti-His antibodies, was conducted to investigate potential variations in protein lysis. “0” means the absence of pronase, while “1:100”, “1:500” and “1:1000” means pronase-to-protein ratio. The “-” symbol indicates the absence of acaricide, while the “+” symbol indicates acaricide added. The presence of enhanced bands (“+” lane) relative to the control conditions, where acaricide was not added (“-” lane), would indicate a binding interaction between the protein and the acaricide. The results reveal that cyflumetofen and AB-1 bind to TcHR96h against degradation, whereas TcHR96h (R448A) and actin do not. The experiments were replicated two times.
(TIF)

S9 Fig Protein densitometry of DARTs. (A) The affinity between *Tc*HR96h and fenpropathrin, cyflumetofen, or AB-1 was tested. The experiments were repeated twice. Lanes 1–5 represent TcHR96h only, TcHR96h + acetone + pronase, TcHR96h + fenpropathrin+ pronase, TcHR96h + cyflumetofen + pronase, and TcHR96h + AB-1 + pronase, respectively. (B) The affinity between *Tc*HR96h (R448A) and fenpropathrin, cyflumetofen, or AB-1 was tested. The experiments were replicated two times. Lanes 1–5 represent *Tc*HR96h (R448A) only, *Tc*HR96h (R448A) + acetone + pronase, *Tc*HR96h (R448A) + fenpropathrin + pronase, *Tc*HR96h (R448A) + cyflumetofen + pronase, and *Tc*HR96h (R448A) + AB-1 + pronase, respectively. *Tc*HR96h was preincubated with various concentrations (10–1000 μM) of cyflumetofen (C) and AB-1 (D) on ice for 1 h first and then at 37°C for 30 min and then digested with Pronase (1:500) for 20 min at 37°C. The experiments were repeated twice. Column charts represent gray values fold relative to “*Tc*HR96h + acetone.” (E) Quantification of the signal intensity ratio between cyflumetofen/actone or AB-1/ acetone under different pronase concentration. The DARTs experiment, employing varying concentrations of pronase along with anti-His antibodies, was conducted to investigate potential variations in protein lysis. “0” means the absence of pronase, while “1:100”, “1:500” and “1:1000” means pronase-to-protein ratio. The “-” symbol indicates the absence of acaricide, while the “+” symbol indicates acaricide added. The presence of enhanced bands (“+” lane) relative to the control conditions, where acaricide was not added (“-” lane), would indicate a binding interaction between the protein and the acaricide. The results reveal that cyflumetofen and AB-1 bind to TcHR96h against degradation, whereas TcHR96h (R448A) and actin do not. The error bars represent the standard errors of two independent experiments.
(TIF)

S1 Table Primer information. (DOCX)

S2 Table The ORF information of TcHR96h. (DOCX)

S3 Table Sequences used for phylogenetic analysis. (DOCX)

S4 Table The similarity between TcHR96h and 8 HR96 genes in *T*. *urticae*. (DOCX)

S5 Table Raw data of cell counts in Fig 5C. (DOCX)
