# Supplementary figures and images for: A detoxification pathway initiated by a nuclear receptor TcHR96h in Tetranychus cinnabarinus (Boisduval)
Source: PLoS Genet. 2023 Sep 14;19(9):e1010911. doi: 10.1371/journal.pgen.1010911 (PMC10501649; doi:10.1371/journal.pgen.1010911)

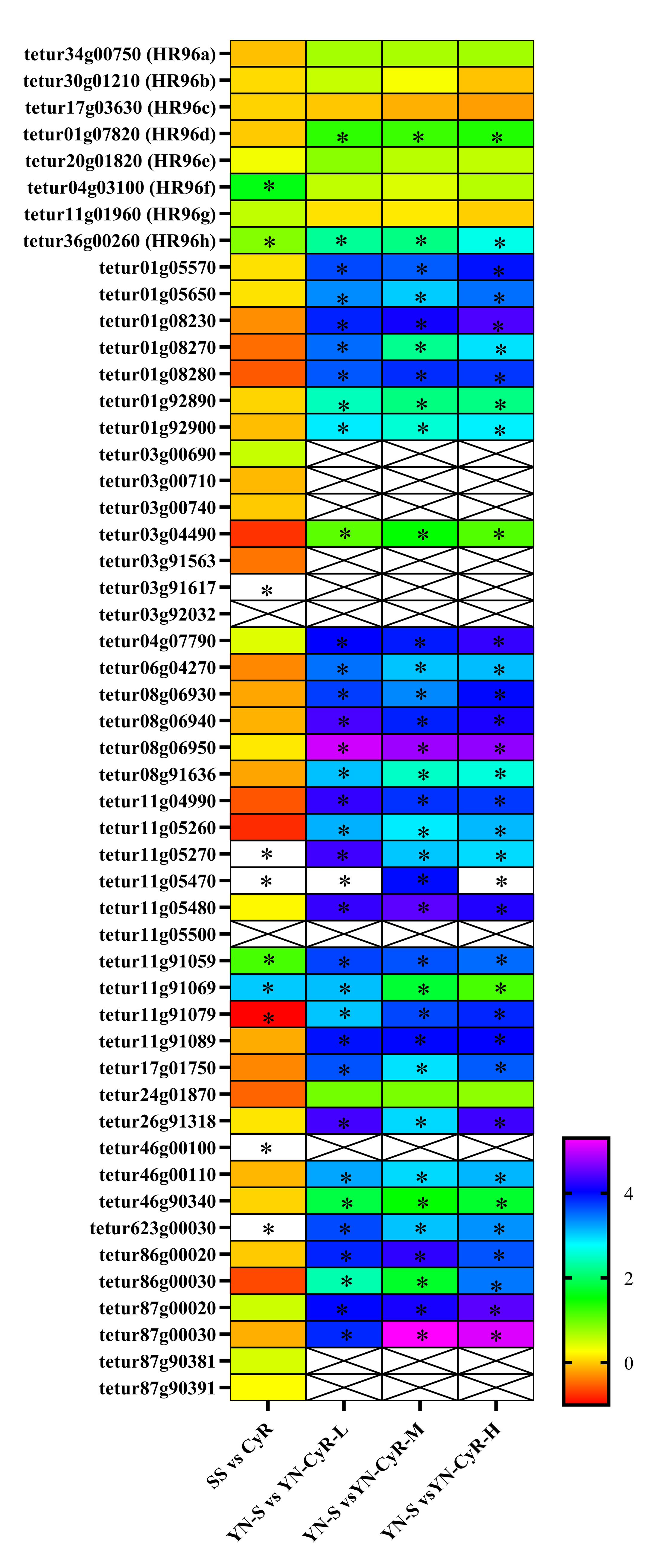

Supplement: S1 Fig — Canonical HR96 (HR96a-h) is marked in the heatplot and the other genes are HR96-like genes. Asterisk represents significant difference (CyR compared with SS or YN-CyR-L/M/H compared with YN-S), |log2 ratio| ≥ 1. The cyflumetofen-resistant strain (YN-CyR) was selected from YN-S which was collected from fields in Yunnan, China by continuous selection with cyflumetofen. The resistance ratio of YN-CyR_L, YN-CyR_M, and YN-CyR_H reached 7.83-, 17.23-, and 86.05-fold, respectively and which were named low (L), medium (M), and high levels of resistance (H). (TIF) [file pgen.1010911.s001.tif]

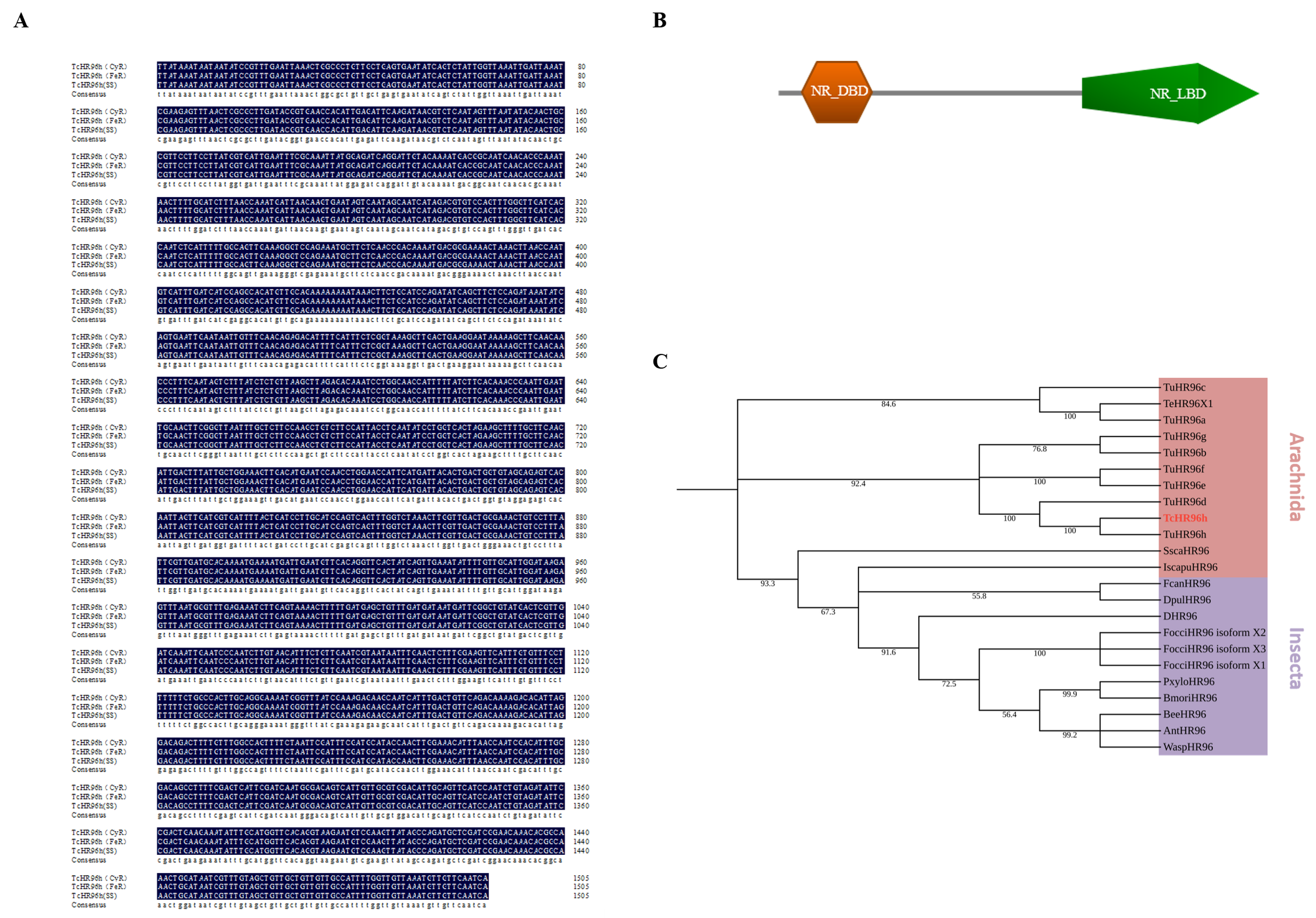

Supplement: S2 Fig — (A) TcHR96h nucleotide sequence comparisons in SS, CyR and FeR strains. (B) Conserved sequence analysis of TcHR96h. NR_DBD indicates DNA binding domain and NR_LBD indicates ligand binding domain. (C) Phylogenetic analyses of TcHR96h. The phylogenetic trees were constructed for amino acid sequences by the maximum likelihood LG + G method. Bootstrap analyses were performed with 1000 iterations, and only bootstrapping values >50 are shown. (TIF) [file pgen.1010911.s002.tif]

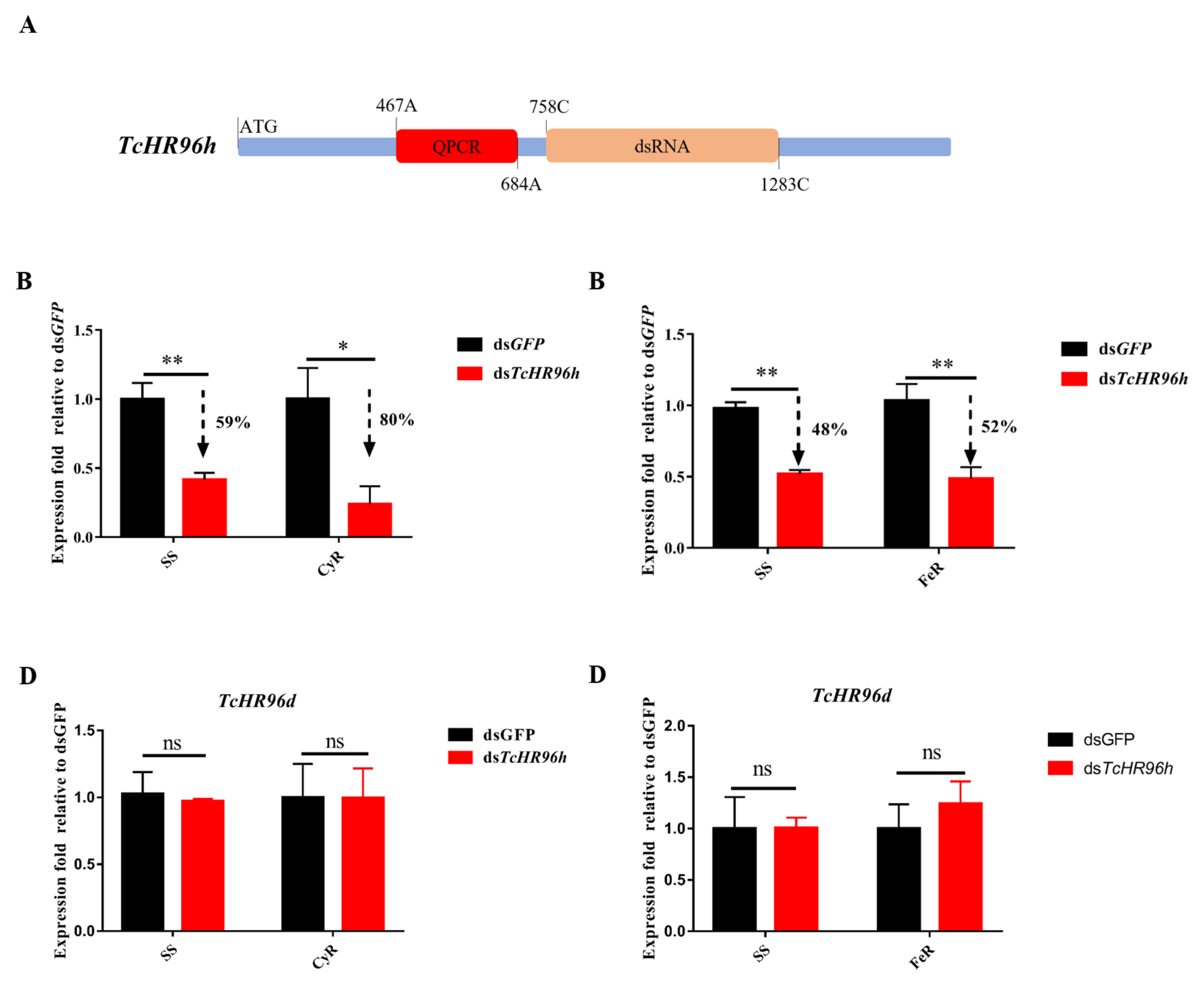

Supplement: S3 Fig — (A) The design of the qPCR and dsRNA fragments. (B) Relative expression of TcHR96h mRNA after RNAi in strains SS and CyR was assessed by qPCR (qPCR: n = 3, mean ± SE, asterisk represents significant difference (dsTcHR96h compared with dsGFP), *P < 0.05, **P < 0.01, two-tailed Student’s t-test). (C) Relative expression of TcHR96h mRNA after RNAi in strains SS and FeR was assessed by qPCR (qPCR: n = 3, mean ± SE, asterisk represents significant difference (dsTcHR96h compared with dsGFP), *P < 0.05, **P < 0.01, two-tailed Student’s t-test). dsGFP, the dsRNA of green fluorescent protein, was used as a negative control. dsTcHR96h, the dsRNA of TcHR96h. Downward arrows indicate decreased expression of TcHR96h, and silencing efficiency is expressed as percentage. (D) Relative expression of TcHR96d mRNA after RNAi in strains SS and CyR was assessed by qPCR (qPCR: n = 3, mean ± SE, ‘ns’ indicates no significant difference between dsTcHR96h and dsGFP, two-tailed Student’s t-test). (E) Relative expression of TcHR96d mRNA after RNAi in strains SS and FeR was assessed by qPCR (qPCR: n = 3, mean ± SE, ‘ns’ indicates no significant difference between dsTcHR96h and dsGFP, two-tailed Student’s t-test). TcHR96d was used to the analyze off-target effects of TcHR96h RNAi. (TIF) [file pgen.1010911.s003.tif]

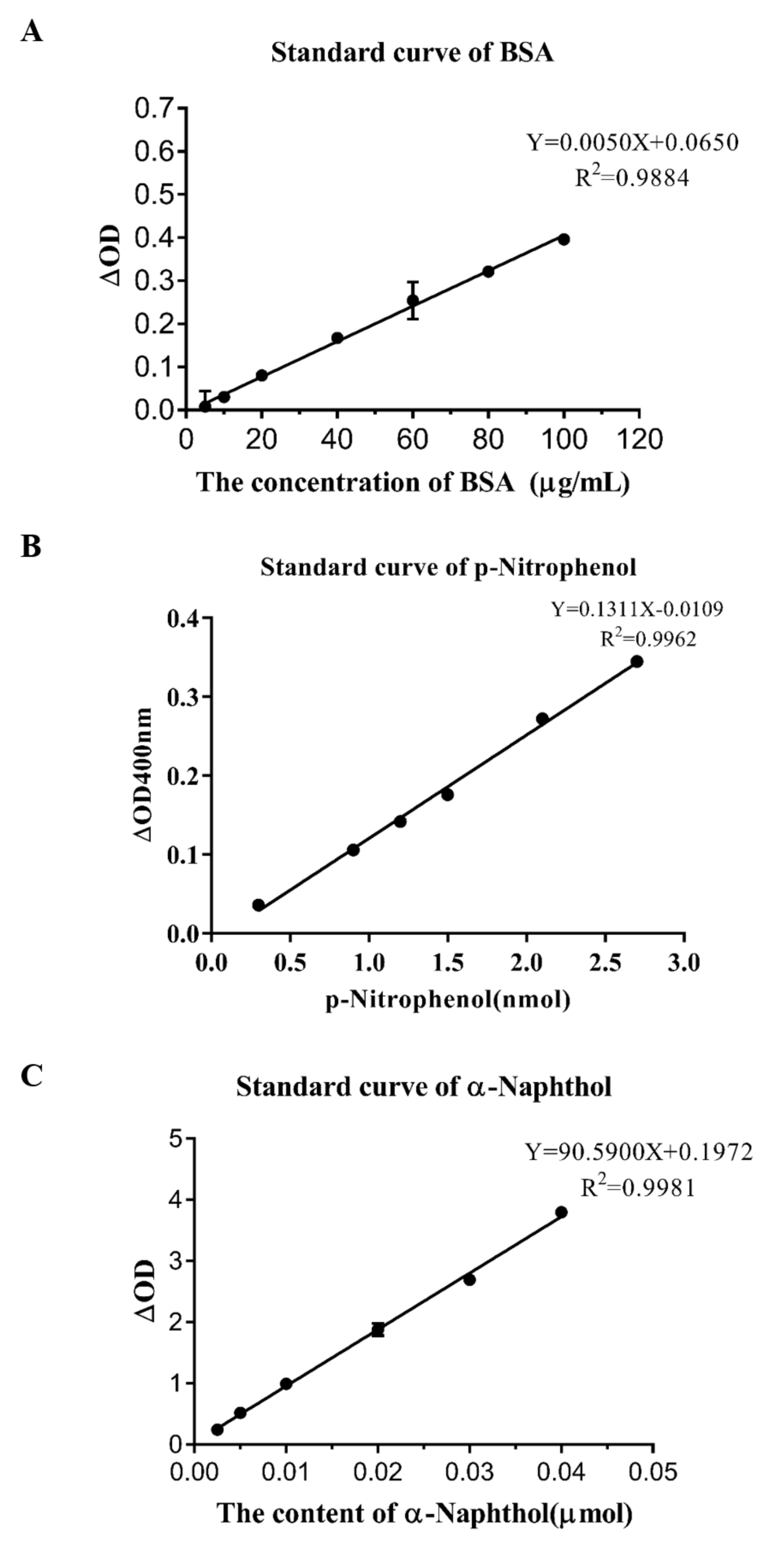

Supplement: S4 Fig — Standard curves of (A) BSA, (B) p-nitrophenol, and (C) of α-naphthol. (TIF) [file pgen.1010911.s004.tif]

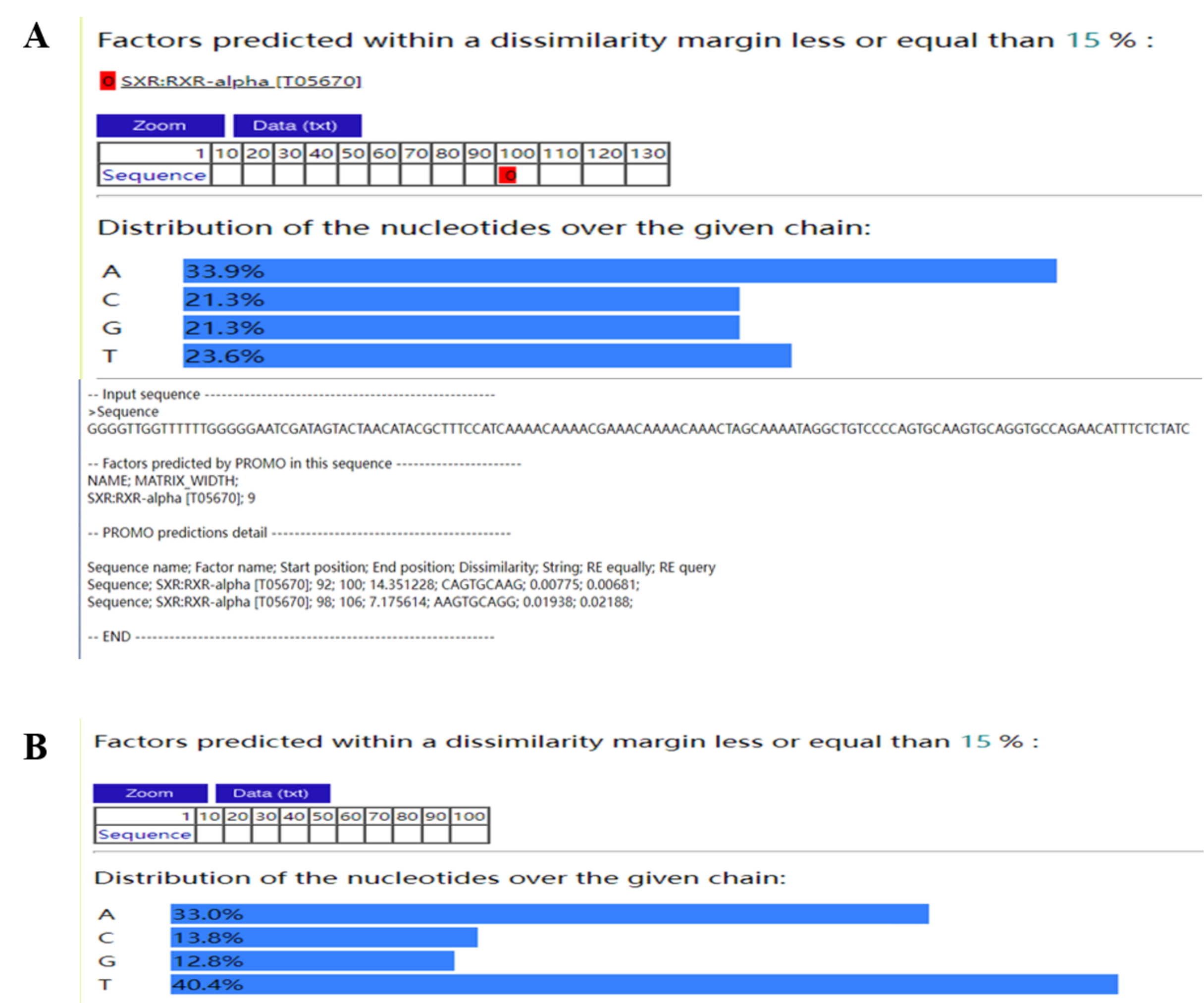

Supplement: S5 Fig — (A) TcGSTm02 promoter truncation from −652 to −545. Only one motif has been predicated. (B) TcGSTm02 promoter truncation from −544 to −430. No motif has been predicated. (TIF) [file pgen.1010911.s005.tif]

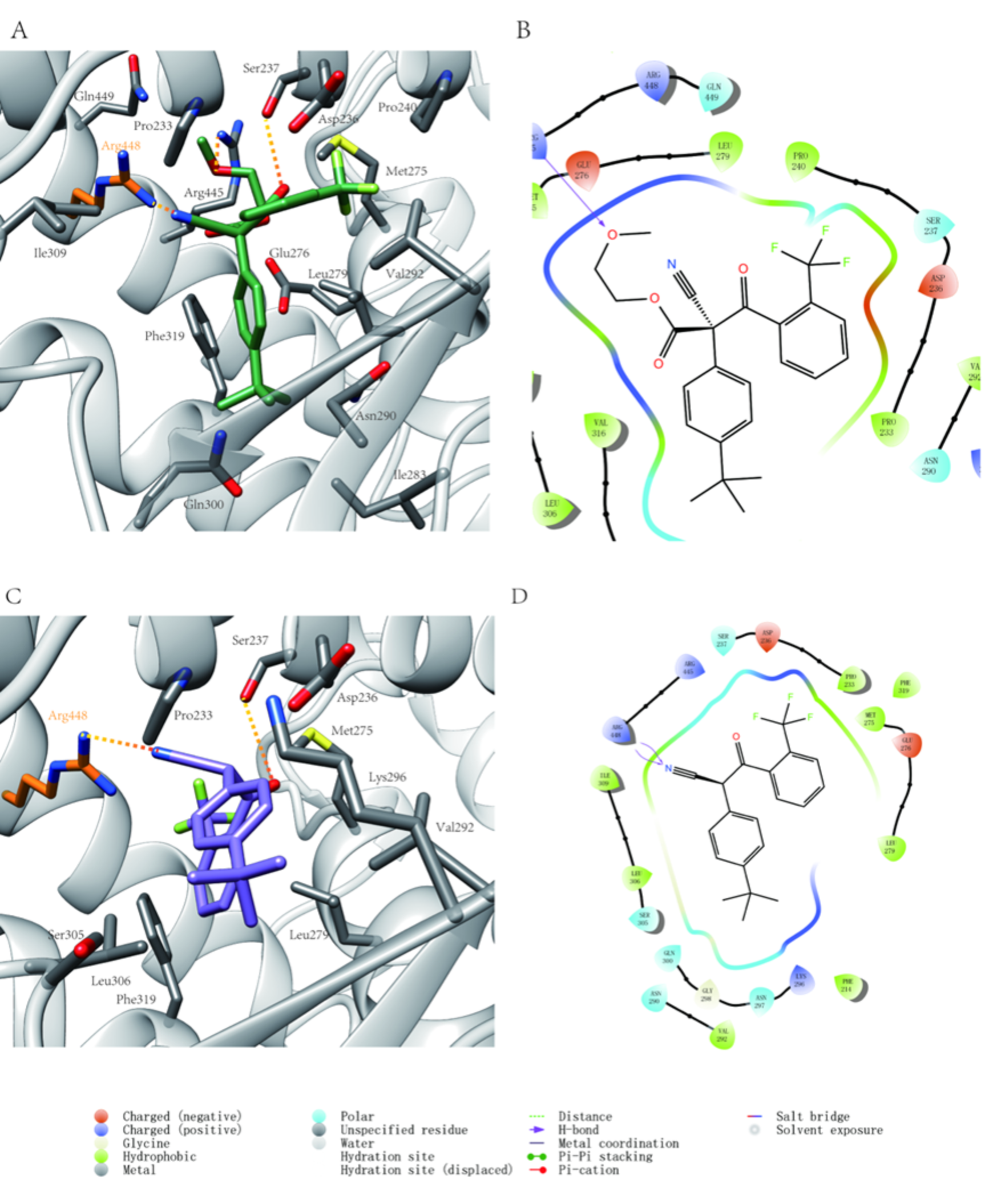

Supplement: S6 Fig — (A) The binding pose of cyflumetofen (green) with TcHR96h. (B) The ligand interaction diagram of cyflumetofen. (C) The binding pose of AB-1 (purple) with TcHR96h. (D) The ligand interaction diagram of AB-1. The side chains of ligand-coordinating residues are displayed and labeled. Hydrogen bonds are indicated using orange dashed lines. (TIF) [file pgen.1010911.s006.tif]

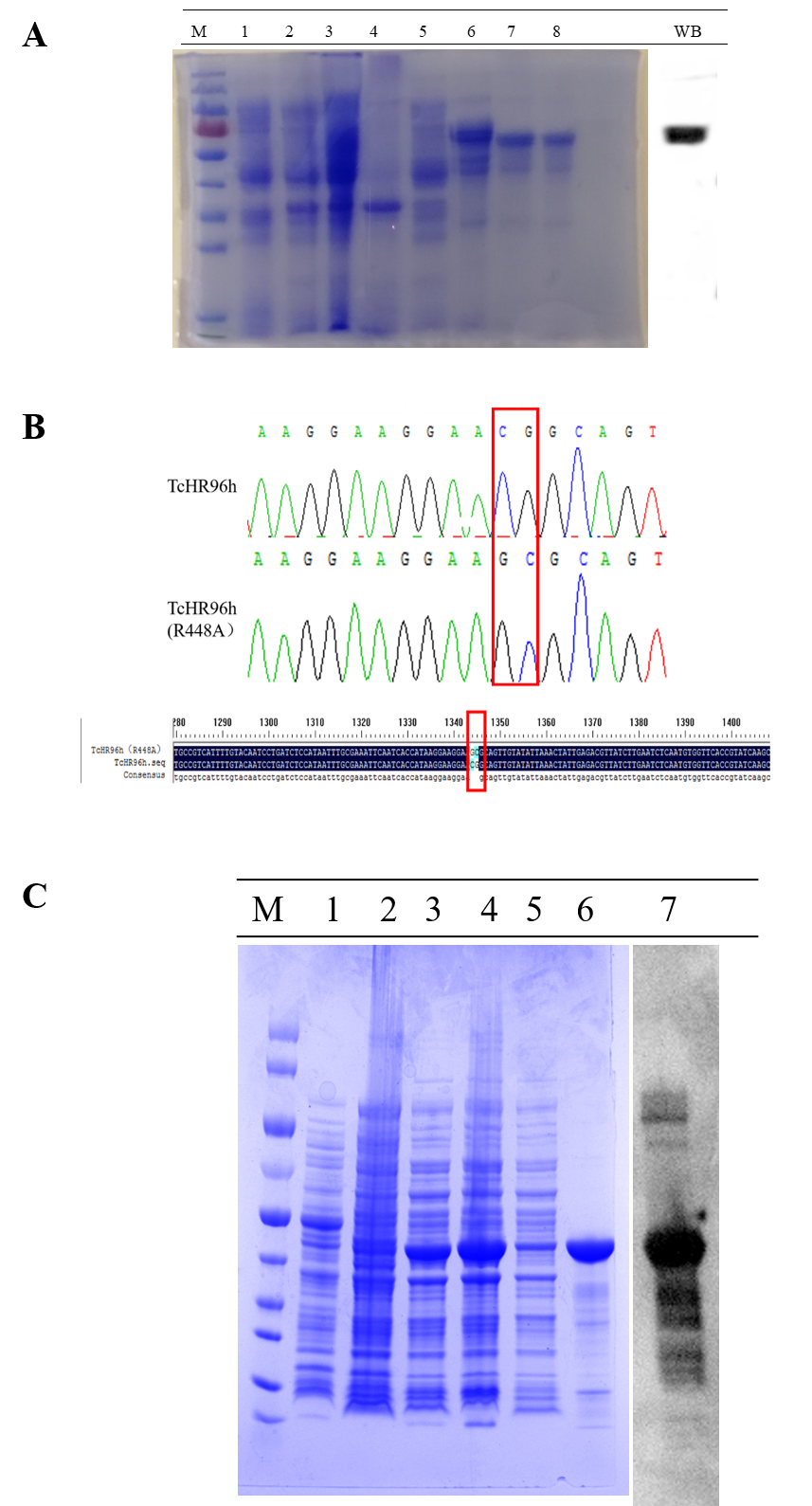

Supplement: S7 Fig — (A) SDS-PAGE analysis of recombinant TcHR96h. Lane M: protein marker, Lane 1: pCold II + IPTG, Lane 2: pCold II::TcHR96h without IPTG, Lane 3: pCold II::TcHR96h + IPTG, Lane 4: supernatant of pCold II::TcHR96h + IPTG, Lane 5: precipitate pCold II::TcHR96h + IPTG, Lane 6: Inclusion bodies denaturing solution, Lane 7: soluble protein Ι from inclusion bodies after renaturation, Lane 8: soluble protein II from inclusion bodies after renaturation, WB: western blotting. (B) Nucleotide sequence alignment between TcHR96h and TcHR96h (R448A). Mutation from G to C at position 1346. (C) SDS-PAGE analysis of recombinant actin. Lane M: protein marker, Lane 1: pCold II + IPTG, Lane 2: pCold II::actin without IPTG, Lane 3: pCold II::actin + IPTG, Lane 4: precipitates of pCold II::actin + IPTG, Lane 5: upernatant pCold II::actin + IPTG, Lane 6: soluble actin protein from inclusion bodies after renaturation, Lane 7: western blotting. (TIF) [file pgen.1010911.s007.tif]

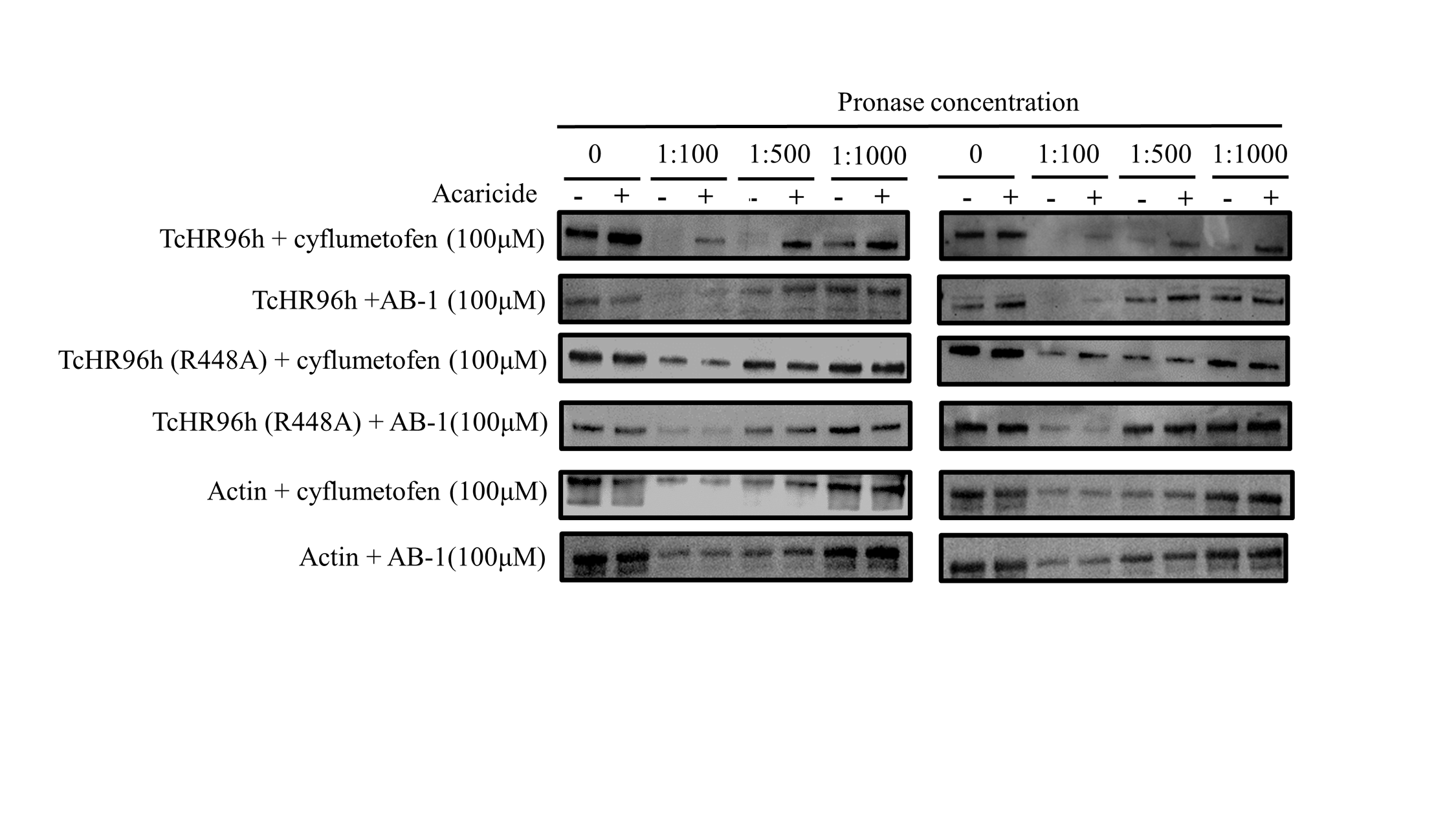

Supplement: S8 Fig — The DARTs experiment, employing varying concentrations of pronase along with anti-His antibodies, was conducted to investigate potential variations in protein lysis. “0” means the absence of pronase, while “1:100”, “1:500” and “1:1000” means pronase-to-protein ratio. The “-” symbol indicates the absence of acaricide, while the “+” symbol indicates acaricide added. The presence of enhanced bands (“+” lane) relative to the control conditions, where acaricide was not added (“-” lane), would indicate a binding interaction between the protein and the acaricide. The results reveal that cyflumetofen and AB-1 bind to TcHR96h against degradation, whereas TcHR96h (R448A) and actin do not. The experiments were replicated two times. (TIF) [file pgen.1010911.s008.tif]

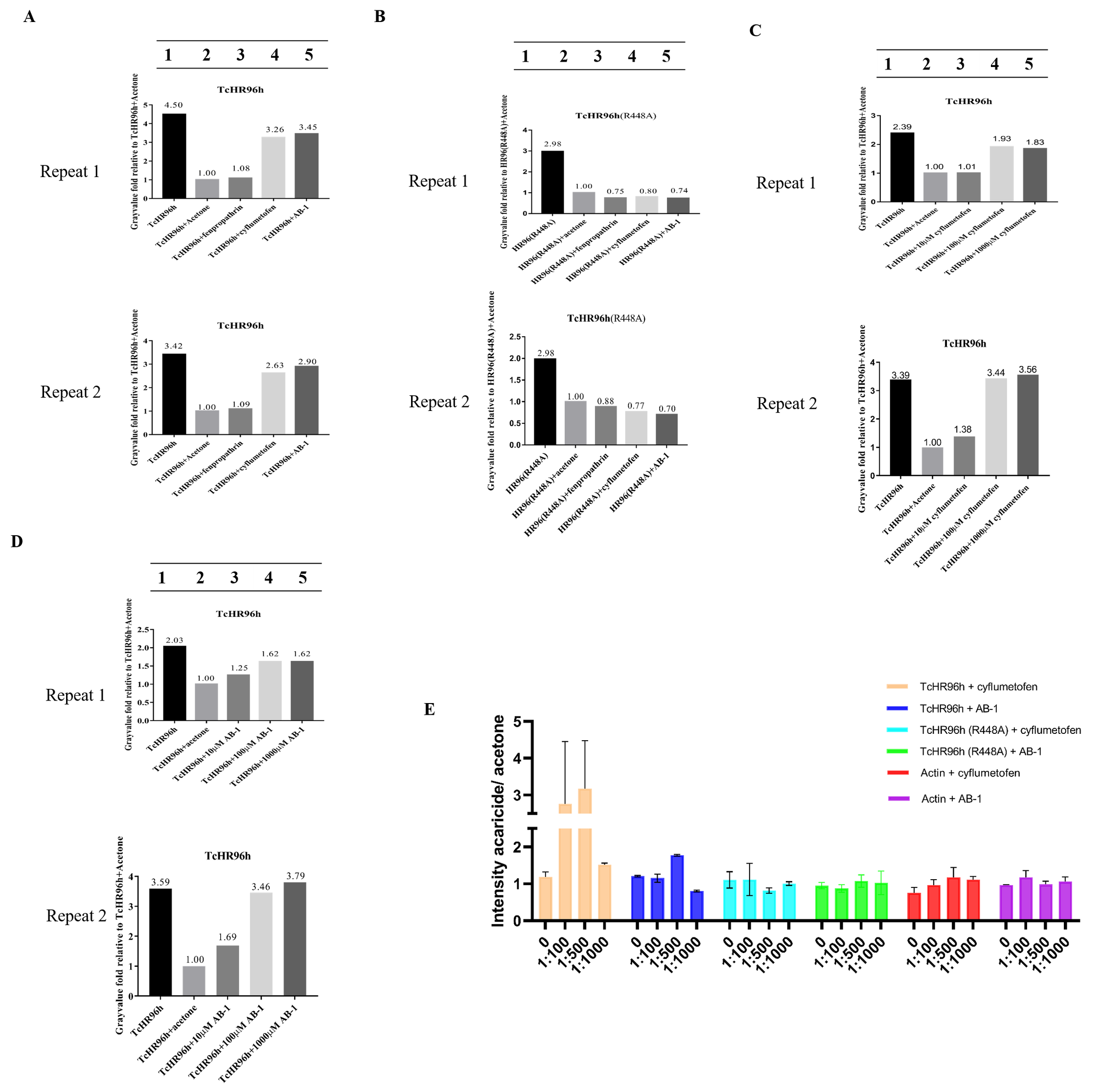

Supplement: S9 Fig — (A) The affinity between TcHR96h and fenpropathrin, cyflumetofen, or AB-1 was tested. The experiments were repeated twice. Lanes 1–5 represent TcHR96h only, TcHR96h + acetone + pronase, TcHR96h + fenpropathrin+ pronase, TcHR96h + cyflumetofen + pronase, and TcHR96h + AB-1 + pronase, respectively. (B) The affinity between TcHR96h (R448A) and fenpropathrin, cyflumetofen, or AB-1 was tested. The experiments were replicated two times. Lanes 1–5 represent TcHR96h (R448A) only, TcHR96h (R448A) + acetone + pronase, TcHR96h (R448A) + fenpropathrin + pronase, TcHR96h (R448A) + cyflumetofen + pronase, and TcHR96h (R448A) + AB-1 + pronase, respectively. TcHR96h was preincubated with various concentrations (10–1000 μM) of cyflumetofen (C) and AB-1 (D) on ice for 1 h first and then at 37°C for 30 min and then digested with Pronase (1:500) for 20 min at 37°C. The experiments were repeated twice. Column charts represent gray values fold relative to “TcHR96h + acetone.” (E) Quantification of the signal intensity ratio between cyflumetofen/actone or AB-1/ acetone under different pronase concentration. The DARTs experiment, employing varying concentrations of pronase along with anti-His antibodies, was conducted to investigate potential variations in protein lysis. “0” means the absence of pronase, while “1:100”, “1:500” and “1:1000” means pronase-to-protein ratio. The “-” symbol indicates the absence of acaricide, while the “+” symbol indicates acaricide added. The presence of enhanced bands (“+” lane) relative to the control conditions, where acaricide was not added (“-” lane), would indicate a binding interaction between the protein and the acaricide. The results reveal that cyflumetofen and AB-1 bind to TcHR96h against degradation, whereas TcHR96h (R448A) and actin do not. The error bars represent the standard errors of two independent experiments. (TIF) [file pgen.1010911.s009.tif]
