# Supplementary material for: A detoxification pathway initiated by a nuclear receptor TcHR96h in Tetranychus cinnabarinus (Boisduval)
Source: PLoS Genet. 2023 Sep 14;19(9):e1010911. doi: 10.1371/journal.pgen.1010911 (PMC10501649; doi:10.1371/journal.pgen.1010911)
Supplement: S1 Table — (DOCX) [file pgen.1010911.s010.docx]

**S1 Table. Primer information.**

| **Gene** | **Forward** | **Reverse** |
| --- | --- | --- |
| qPCR-*TcGSTm01* | CAACGTATTGACCAGTTGTG | CGCCAAGAAATTGAGCCAATG |
| qPCR-*TcGSTm02* | TCGGATGTTGTTGGCTCAAG | TCATCATCGATGTAGTACGG |
| qPCR-*TcGSTm03* | TTCGTTTTGGGTGCAAAGG | AGCACCAATGTTTGGTAAGGC |
| qPCR-*TcGSTm04* | ATTGGCACTATTCCGAGCC | CAGGTTTTCATGTGGGTCC |
| qPCR-*TcGSTm05* | ACTCAAGCAAGCTGACCAAC | TCAACGTACCAAGCTGACCA |
| qPCR-*TcGSTm06* | ACGGCTACTTTTGGCTCATAC | ACCAATCCATGTTTCCGAGC |
| qPCR-*TcGSTm07* | CAAGTTCATCCTCGGTGGTC | TTGATTTCGGGCAAGGCTTC |
| qPCR-*TcGSTm08* | GTTCGAGGTGTTGGTGAACC | AGCCACTCCGATTTGTCGTA |
| qPCR-*TcGSTm09* | CAAGAAATGCCCTGGAATC | GGAAGCTAAGCCAGGTCTATCGAAA |
| qPCR-*TcGSTm10* | GTCATTTCTCGGTCCTAA | GTTCGTCGCTACAAACTC |
| qPCR-*TcGSTm11* | AACAAGCTGACCAACCCCTC | TCAAGAACGTCCTCGGTGTG |
| qPCR-*TcGSTm12* | GGCTCACACTGGTCAAGAGTA | CAGGTTCGGGTGCAAGTGA |
| qPCR-*TcHR96a* | AGCGGACTGATAACCCTTCG | AAGGATCGAGTGCAGCAGAG |
| qPCR-*TcHR96b* | GCTTGCCCGTTCAATGACAA | GACGGACGATCGTTGGTTTG |
| qPCR-*TcHR96c* | TGACGATGAATGTTGGCAGG | GCCCAATAGCTTCGGTTCCA |
| qPCR-*TcHR96d* | GTCGCAAGTGTCGTTTGGTT | GGTTGTCTTGAGCATGGCAG |
| qPCR-*TcHR96e* | AAGATTACTCGGCGCCATTGT | AGGTTTCGTTGGAAGAGGCA |
| qPCR-*TcHR96f* | TTCCTCTGCCCTTTTACGGA | TGCAATGCAGCTTCATCCAAC |
| qPCR-*TcHR96g* | TGCTCAAGCCACGTCTTCAT | AGCACAAATTTGGCGAAGGT |
| qPCR-*TcHR96h* | AACGAGTCAAACAGCCGAAT | TGATCCTTGCATCGAGTCAG |
| qPCR -*a-tubulin* | ACTACGCTCGTGGCCACTATACAA | ACCAGATCCAGTTCCACCTCCAAA |
| qPCR -*RP18S* | ACGTGCTGGTGAACTTACCGAAGA | TGCCTATTCAAGAACCAAAGTGGG |
| *TcHR96h*-full | ATGATTGAACAACATTT | TTATAAATAATAATATCCGT |
| *TcHR96h*-RNAi | taatacgactcactataggg  -CATGTCAACTTTCCAGCAATA | taatacgactcactataggg  -GCAATCAACACGCAAATAAC |
| *GFP*-RNAi | taatacgactcactataggg-  -CAGTTCTTGTTGAATTAGATG | taatacgactcactataggg  -TTTGGTTTGTCTCCCATGATG |
| pCold II- *TcHR96h* | tcatcatcatcatca-  -ATGATTGAACAACATTTAACAACCA | aggtcgacaagcttg  -TTATAAATAATAATATCCGTTTGAA |
| pCold II- *actin* | catcatcatatggagctcggtacc  -ATGTGTGACGACGAAGTAGCA | tctagactgcaggtcgacaagctt-TTAGAAACACTTTCTGTGGACAA |
| *TcGSTm02*-promoter | GGGGTTGGTTTTTTGGGGGAATCGA | GGCGTCTTCCATGGTGGCTT |
