# Supplementary material for: A detoxification pathway initiated by a nuclear receptor TcHR96h in Tetranychus cinnabarinus (Boisduval)
Source: PLoS Genet. 2023 Sep 14;19(9):e1010911. doi: 10.1371/journal.pgen.1010911 (PMC10501649; doi:10.1371/journal.pgen.1010911)
Supplement: S2 Table — (DOCX) [file pgen.1010911.s011.docx]

**S2 Table. The ORF information of TcHR96h.**

| **Gene** | **ORF(bp)** | **Anmino acid** | **Molecular weight(KDa)** | **Isoelectric point** |
| --- | --- | --- | --- | --- |
| *TcHR96h* | 1506 | 501 | 56.88 | 6.55 |
