# Supplementary material for: A detoxification pathway initiated by a nuclear receptor TcHR96h in Tetranychus cinnabarinus (Boisduval)
Source: PLoS Genet. 2023 Sep 14;19(9):e1010911. doi: 10.1371/journal.pgen.1010911 (PMC10501649; doi:10.1371/journal.pgen.1010911)
Supplement: S3 Table — (DOCX) [file pgen.1010911.s012.docx]

**S3 Table. Sequences used for phylogenetic analysis**

| **Gene name** | **Species** | **Sequence ID** |
| --- | --- | --- |
| TuHR96a | *Tetranychus urticae* | XP_015793504.1 |
| TuHR96b | *Tetranychus urticae* | XP_015786656.1 |
| TuHR96c | *Tetranychus urticae* | XP_015781631.1 |
| TuHR96d | *Tetranychus urticae* | XP_015790255.1 |
| TuHR96e | *Tetranychus urticae* | XP_015783958.1 |
| TuHR96f | *Tetranychus urticae* | XP_025017423.1 |
| TuHR96g | *Tetranychus urticae* | XP_015792611.1 |
| TuHR96h | *Tetranychus urticae* | XP_025018141.1 |
| TeHR96X1 | *Tetranychus evansi* | AYV89225.1 |
| SscalHR96 | *Sarcoptes scabiei* | KAF7491292.1 |
| PxyloHR96 | *Plutella xylostella* | XP_037967618.1 |
| WaspHR96 | *Nasonia vitripennis* | XP_001606458.1 |
| IscapuHR96 | *Ixodes scapularis* | XP_029831621.2 |
| FocciHR96X1 | *Frankliniella occidentalis* | XP_026282509.1 |
| FocciHR96X2 | *Frankliniella occidentalis* | XP_026282511.1 |
| FocciHR96X3 | *Frankliniella occidentalis* | XP_026282508.1 |
| FcanHR96 | *Folsomia candida* | XP_035713289.1 |
| DpulHR96 | *Daphnia pulex* | EFX89804.1 |
| DHR96 | *Drosophila melanogaster* | AAC46928.1 |
| AntHR96 | *Camponotus floridanus* | EFN64539.1 |
| BmoriHR96 | *Bombyx mori* | XP_004933007.1 |
| BeeHR96 | *Apis mellifera* | XP_624213.3 |
