# Supplementary material for: A detoxification pathway initiated by a nuclear receptor TcHR96h in Tetranychus cinnabarinus (Boisduval)
Source: PLoS Genet. 2023 Sep 14;19(9):e1010911. doi: 10.1371/journal.pgen.1010911 (PMC10501649; doi:10.1371/journal.pgen.1010911)
Supplement: S4 Table — (DOCX) [file pgen.1010911.s013.docx]

**S4 Table. The similarity between TcHR96h and 8 HR96 genes in *T. urticae***

|  | Similarity (*TcHR96h*) |
| --- | --- |
| *TuHR96a* | 23.27% |
| *TuHR96b* | 18.26% |
| *TuHR96c* | 20.54% |
| *TuHR96d* | 32.98% |
| *TuHR96e* | 18.30% |
| *TuHR96f* | 20.30% |
| *TuHR96g* | 19.90% |
| *TuHR96h* | 95.42% |
