# Supplementary material for: A detoxification pathway initiated by a nuclear receptor TcHR96h in Tetranychus cinnabarinus (Boisduval)
Source: PLoS Genet. 2023 Sep 14;19(9):e1010911. doi: 10.1371/journal.pgen.1010911 (PMC10501649; doi:10.1371/journal.pgen.1010911)
Supplement: S5 Table — (DOCX) [file pgen.1010911.s014.docx]

**S5 Table. Raw data of cell counts in Fig.7. C**

| **Trentment** | **Repeat** | **Total** | **Positive** |
| --- | --- | --- | --- |
| EGFP+ DMSO | 1 | 10 | 0 |
|  | 2 | 7 | 0 |
|  | 3 | 15 | 2 |
| TcHR96h+ DMSO | 1 | 10 | 0 |
|  | 2 | 20 | 1 |
|  | 3 | 10 | 2 |
| EGFP+ Cyflumetofen | 1 | 14 | 2 |
|  | 2 | 10 | 1 |
|  | 3 | 20 | 0 |
| TcHR96h+Cyflumetofen(30min) | 1 | 8 | 4 |
|  | 2 | 13 | 6 |
|  | 3 | 15 | 9 |
| TcHR96h+ Cyflumetofen(6 h) | 1 | 37 | 32 |
|  | 2 | 22 | 19 |
|  | 3 | 28 | 25 |
| TcHR96h+ AB-1 (6 h) | 1 | 38 | 38 |
|  | 2 | 36 | 33 |
|  | 3 | 29 | 25 |
